# Leaf Morphology, Taxonomy and Geometric Morphometrics: A Simplified Protocol for Beginners

**DOI:** 10.1371/journal.pone.0025630

**Published:** 2011-10-03

**Authors:** Vincenzo Viscosi, Andrea Cardini

**Affiliations:** 1 Museo Erbario del Molise, Dipartimento di Scienze e Tecnologie per l'Ambiente e il Territorio, Università del Molise, Contrada Fonte Lappone, Pesche, Italy; 2 Dipartimento di Biologia, Universitá di Modena e Reggio Emilia, Modena, Italy; 3 Hull York Medical School, The University of Hull, Hull, United Kingdom; 4 Centre for Forensic Science, The University of Western Australia, Crawley, Western Australia, Australia; Institut de Biologia Evolutiva - Universitat Pompeu Fabra, Spain

## Abstract

Taxonomy relies greatly on morphology to discriminate groups. Computerized geometric morphometric methods for quantitative shape analysis measure, test and visualize differences in form in a highly effective, reproducible, accurate and statistically powerful way. Plant leaves are commonly used in taxonomic analyses and are particularly suitable to landmark based geometric morphometrics. However, botanists do not yet seem to have taken advantage of this set of methods in their studies as much as zoologists have done. Using free software and an example dataset from two geographical populations of sessile oak leaves, we describe in detailed but simple terms how to: a) compute size and shape variables using Procrustes methods; b) test measurement error and the main levels of variation (population and trees) using a hierachical design; c) estimate the accuracy of group discrimination; d) repeat this estimate after controlling for the effect of size differences on shape (i.e., allometry). Measurement error was completely negligible; individual variation in leaf morphology was large and differences between trees were generally bigger than within trees; differences between the two geographic populations were small in both size and shape; despite a weak allometric trend, controlling for the effect of size on shape slighly increased discrimination accuracy. Procrustes based methods for the analysis of landmarks were highly efficient in measuring the hierarchical structure of differences in leaves and in revealing very small-scale variation. In taxonomy and many other fields of botany and biology, the application of geometric morphometrics contributes to increase scientific rigour in the description of important aspects of the phenotypic dimension of biodiversity. Easy to follow but detailed step by step example studies can promote a more extensive use of these numerical methods, as they provide an introduction to the discipline which, for many biologists, is less intimidating than the often inaccessible specialistic literature.

## Introduction

Leaf morphology is central to plant taxonomy and systematics [1] and it has mostly been studied using traditional morphometrics [2], [3]. In the last decade, however, there has been an increasing interest in the use of modern geometric morphometrics (GMM) to study the form of leaves. GMM analyzes the relative positions of anatomical landmarks and sets of points used to approximate curves (outlines) and surfaces to quantify size and shape [3]. The geometric information of shape differences is preserved, statistical power is increased [4], patterns can be visualized using image rendering and a variety of other diagrams [5]. The increase in the number of publications using GMM within [5], [6] and from outside [7], [8] biology has been exponential and pays testament to the success of this set of methods.

Taxonomists and botanists have recognized the potential of GMM in their field: “If the systematist is really interested in focusing on shape, separately from size, and/or on testing hypotheses about shape differences, then traditional approaches are not adequate; landmark methods are clearly superior, especially when the landmarks represent well-defined, biologically homologous points “… there is no information in the context of a set of landmarks that cannot be extracted by application of the … approach” ([3] p. 667–669). Leaf shape variability has been investigated using analyses of landmarks and outlines to accurately discriminate species and their hybrids. For instance, using GMM on leaves, Jensen [9] and Jensen et al. [10] detected hybridization in black and red American oaks and Peñaloza-Ramirez et al. [11] demonstrated that oak hybrids and backcrosses have intermediate morphology. Viscosi et al. [12], [13] also applied GMM and found evidence that in European white oaks leaf shape correlates strongly with the taxonomy of species and hybrids inferred using molecular data. In taxonomy and other fields, genetics and morphometrics can fruitfully interact as complementary tools to understand the origin of phenotypic differences [14].

This type of analyses, however, has mostly focused on insects and mammals and has not yet been extensively performed in botany. Only recently GMM studies on the effects of the environment on the development of plants have begun to gain precedence in the literature: Albarrán-Lara et al. [15] examined fluctuating asymmetry in hybrids of two inter-fertile Mexican white oaks; Viscosi et al. [13] demonstrated a correlation between the deepness of leaf lobes and temperature, rainfall and, less frequently, altitude in European white oaks; Van der Niet et al. [16] explored the covariation of shape with pollinators using analyses of three-dimensional data from computerized reconstructions of *Satyrium* flowers based on micro-computed tomography scanning. Van der Niet et al. [16] provides an especially good example of the analytical power of GMM. Using a sophisticated technology for accurate data collection and visualization in combination with principal component analysis, a simple statistical method to summarize shape variables, effectively illustrated that floral shape is associated with pollinator classes. Their findings mirror those of a previous series of GMM studies by Gómez et al. [17]–[19] on the genetics and selective pressures behind the observed variation in *Erysimum* flowers. Gómez and colleagues not only suggested that pollinators strongly select corolla shape by choosing flowers with high reward, but they were also able to add details to this story showing that: “(1) Interactions with generalist organisms may produce strong selection. (2) Spatial changes in main pollinators result in divergent selection across populations. (3) Geographic mosaics depend on a balance between mutualistic and antagonistic selection. (4) Selection mosaics operate at fairly small spatial scales” (p. 245, [18]).

This rapid overview with some examples of applications of GMM in biology and particularly in botany suggests that the method is more than promising and has already proved its effectiveness in numerous studies. Despite this, botany has lagged behind zoology in fully exploiting GMM. A quick but very crude estimate of the difference in terms of publication output can be obtained using Google Scholar to search either “geometric morphometrics zoology” or “geometric morphometrics botany”. The first search returns about five times more results than the second one. In both fields, most of the studies concern taxonomic questions. As most identifications are still largely based on morphology, this is somewhat unsurprising. Taxonomists should indeed be especially keen on taking advantage of new quantitative methods for the description of form. Technological and methodological advancements may soon provide more efficient ways of detecting biodiversity and discriminating taxonomic groups using shape data [20], [21] including semi-automated computerized tools [22].

This paper is aimed at scientists who have little or no experience of GMM and would like to understand if and how it might be applied to taxonomy and botany. We will utilise user-friendly freeware software to provide a step-by-step example on how to:

- measure population variation in size and shape of leaves using Cartesian coordinates of anatomical landmarks and Procrustes based GMM;
- test group differences by partitioning variance into components (population, tree, leaf and measurement error) which are statistically compared in a hierarchical way (i.e., to assess if population differences are larger than differences among trees of the same populations, and whether these are larger than those among leaves of the same tree etc.);
- visualize leaf shape using diagrams (e.g., rendering of outlines, wireframes and thin plate spline deformation grids).

This study will, we hope, stimulate beginners to explore the potential of GMM in botany and facilitate its use in taxonomy. Indeed, an accurate quantification and effective visualization of the main levels of morphological variation in leaves, flowers and other structures is key to gaining insight into the evolutionary and ecological processes of phenotypic diversification and provides the fundamental basis from which to develop more complex studies for achieving “new perspectives on the interplay of phenotype, genotype and environment ... [and a] better understanding of ontogenetic and phylogenetic processes” in plant variation (p. 669, [3]).

## Materials and Methods

### Ethics Statement

No specific permits were required for the described field studies: a) no specific permissions were required for these locations/activities; b) location are not privately-owned or protected; c) the field studies did not involve endangered or protected species.

### Plant material

The main level of the comparison is geographic samples, which, for simplicity, we will loosely refer to as ‘populations’. We used a perfectly balanced design with the same number of observations within each group. This is desirable as it facilitates computations and avoids giving greater weight to groups with larger samples. For each of the two populations, situated approximately 1.5 km apart, two leaves of sessile oak (*Quercus petraea* (Mattuschka) Liebl., 1784) were sampled at random from 22 randomly selected trees. Localities of provenance are near the municipality of Campobasso and Busso (Table 1), which we will use henceforth as the names for the populations. Species assignment was verified using microsatellite genetic data on samples from three sympatric white oak species including the study populations [23].

**Table 1:** Sample localities and size.

*population*	*geographic coordinates*	*N(trees)*	*N(leaves)*
Campobasso	41.5513; 14.6171	22	44
Busso	41.5587; 14.5954	22	44

### Landmark configuration

Leaves were pressed, dried and scanned with the abaxial surface uppermost using an Epson GT-15000 scanner with a resolution of 300 dpi. The entire data collection procedure (i.e., image acquisition and landmark digitization) on the sample of leaves was repeated twice to estimate measurement error. The repetition was performed two weeks after the first round of data collection.

**Figure 1:**
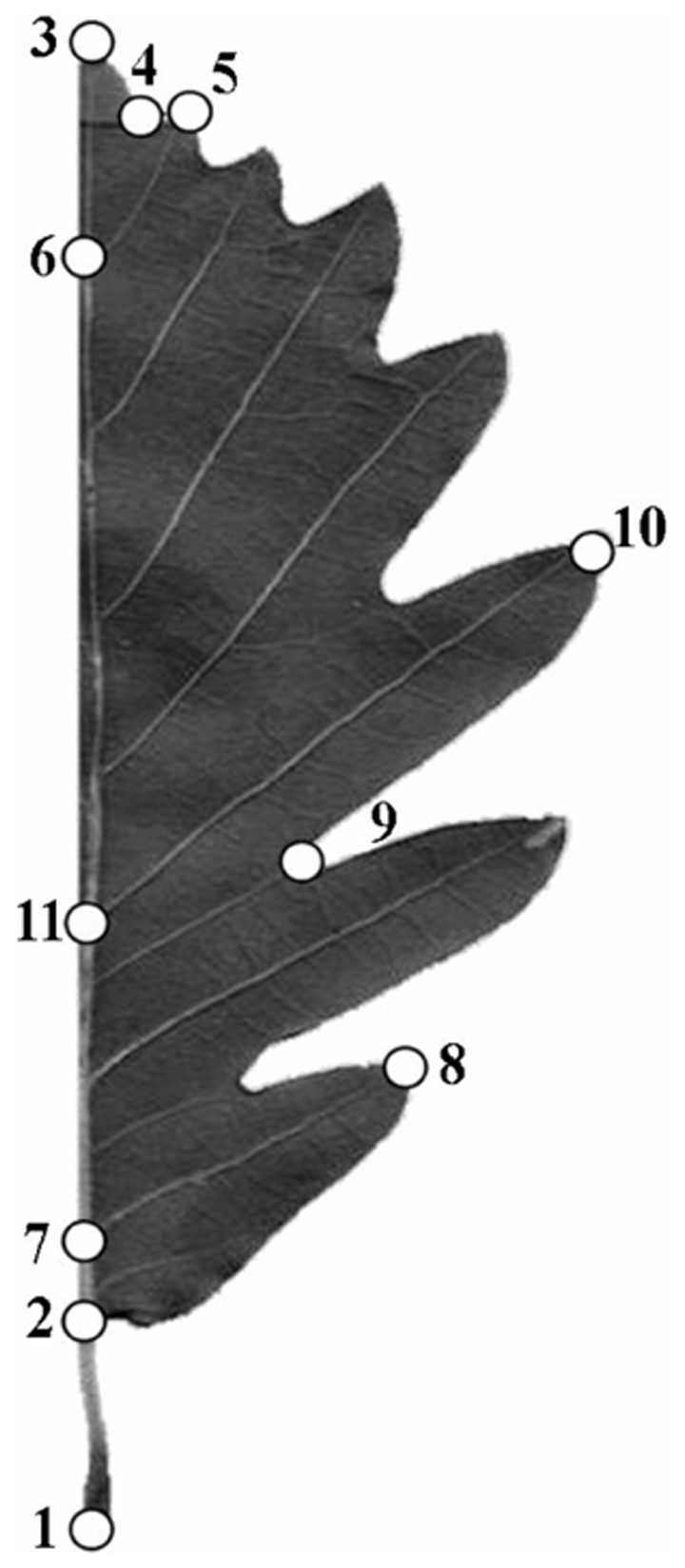
Landmark configuration. From Viscosi et al., 2009, modified.

Scanned images were used to record 11 landmarks on the right half of each leaf (Figure 1 – see Viscosi et al. [12], for landmark definitions). We focused on one side only to adopt the same configuration as used in previous studies on the same species [12], [13]. This is a common expedient to reduce the time of data collection in symmetric structures. However, if patterns of asymmetry and/or data on both sides of the leaf are required, such analyses can be performed in MorphoJ [24] using the methods described in Klingenberg et al. [25].

### Step by step geometric morphometric and statistical analyses

#### 1a) From landmark configurations to shape variables: theoretical background

Landmark based GMM [4], [5], [26] captures the form of a structure using Cartesian coordinates of a configuration of points. These points must have a one to one correspondence in the specimens to be compared. The type of correspondence (topographical, anatomical, developmental etc.) depends on the scientific questions being asked ([27]; see also Discussion). There is no absolute landmark configuration on any given structure and the choice of the configuration must be led by a clear statement on the hypotheses being tested: “... in a study of bat and bird wings if one is interested in function, landmarks at wing tips and along the leading and trailing edges may be functionally equivalent; they might embody the question in being related to functionally relevant aspects of form. However, these landmarks may lie on structures that are not equivalent in other ways; for a study of growth or evolution, alternative landmarks may be the most suited ones” [p. 89, 27]. The choice of landmarks is therefore a crucial step in the analysis and the use of outline methods, equally spaced points on contours and surfaces (called semilandmarks) [5] or other techniques, which do not depend on the explicit identification of anatomical landmarks, does not avoid the fundamental assumption of correspondence of the morphometric descriptors being used [28]. They, at least in their current form, simply delegate to an algorithm the role of the anatomist in selecting where to exactly locate the points [28]. Despite the apparent rigour, objectivity and ability to extract information otherwise difficult to obtain, different mathematical criteria and selection of parameters (e.g., the densitiy of semilandmarks) may produce incongruent shape distances and results [28], [29]. More importantly they leave the question about the biology underlying algorithmically defined anatomical features open and the possibility that mathematically corresponding points might actually map onto areas inconsistent among individuals and in relation to the biological model set by the scientific questions being addressed is real and should be acknowledged [27].

GMM is a disparate set of techniques [5] with a common purpose: the statistical analysis of differences in form using a quantitative description that preserves the geometry of shape variation. Unless differently specified, we will henceforth use GMM to refer only to landmark-based methods using Procrustes analysis. This is currently the standard method for the analysis of landmark data and the most common GMM technique together with various forms of Fourier analysis for the study of outlines [6], [30]. Thus, we performed a generalized Procrustes analysis (GPA, [31]) to separate size and shape components of form variation. Shape coordinates were computed by standardizing each configuration to unit centroid size and by minimizing differences in translation and rotation of all specimens using a least-square algorithm. Size was measured for each specimen as the centroid size of the landmark configuration. The centroid size (to which we will refer to simply as size throughout much of this article) measures the dispersion of landmarks using a function of their distances from the centroid, which is the ‘baricenter’ of a configuration: scattered landmarks will have a large centroid size, clumped landmarks a small one. This series of operations used to compute size and shape variables from the raw Cartesian coordinates of landmarks, commonly described as GPA, is also called Procrustes superimposition / alignment / registration / fit and it is one of several superimposition methods. Compared to other methods, the GPA has desirable statistical properties as a higher power in tests and increased accuracy in estimating sample means [32]–[34].

Procrustes shape coordinates are redundant. This means that there are more coordinates than the actual number of shape variables after the superimposition. In two dimensional analyses (i.e., landmarks on flat images, as in our case study), four degrees of freedom are lost: one for size standardization, two for translating configurations on the X and Y coordinate axes to superimpose their centroids, another one for minimizing rotational differences. In three-dimensions, when each landmark has an X, Y and Z coordinate, the loss of degrees of freedom is computed in the same way but the total amounts to seven due to an extra axis (Z) for translation and two more planes for rotations. Multivariate parametric statistical tests may, depending on the way they were implemented by the software authors, incorrectly compute variable degrees of freedom if performed on shape coordinates. The redundancy is, however, readily identified and accounted for by performing a principal component analysis (PCA) using the variance-covariance matrix of the GPA shape coordinates. A PCA is a way to summarize multivariate data by building linear combinations of the original variables that are uncorrelated and maximize the sample total amount of variance explained [2]–[35]. The spatial relationships between specimens are unaltered, the whole set of PCs accounts for the entire variance in the original variables and nothing is changed in the structure of the data, as only the axes on which they are projected have been rigidly rotated. The specimens can be thought of as a cloud of points in a multivariate space where the observer has changed his/her position to get a better view of the longest sides of the cloud. In GMM a PCA on shape variables is occasionally referred to as a relative warp analysis [36], [37]. There is a subtle difference between the two methods; however, in virtually all biological applications, they effectively function in the same manner and produce identical scores. For this reason that we favoured the well know term PCA.

#### 1b) From landmark configurations to shape variables: software applications and shape spaces

We used the freeware program MorphoJ [24] for most of the analyses. The program is concisely presented in Klingenberg [24], but also has an extensive html user's guide. We will spend some time in detailing the specific operations performed in MorphoJ and some of the other freeware software we used. These programs are powerful comprehensive computer packages, which can perform a variety of analyses and data manipulations. Several others, which we are not using, can be found following the links at http://life.bio.sunysb.edu/morph/ (Accessed 2011 June 8). For more flexibility and a broader spectrum of analytical tools, shape data can be imported in R (www.r-project.org/. Accessed 2011 June 8) or directly generated in this statistical environment by using the package ‘shapes’ [38]. Claude's book [39] on GMM in R provides guidelines and examples to assist with using this software language.

Files for data manipulation and digitization were created mostly using software from the TPS Series, a suite of programs for two-dimensional geometric morphometric analyses [37]:

First we created a TPS file in TPSUtil using the “build TPS file from images” option. This is a simple ascii text file with the extension .TPS, which lists the names of the image files and specifies that no landmarks have yet been digitized. TPS files can be manipulated in TPSUtil (e.g., to change the order of / remove landmarks or specimens etc.) or manually in any text editor.

Then we opened the TPS file in TPSDig and digitized the 11 landmarks in the same order on each picture, after setting a scale factor. The scale factor was set using the image tool menu of TPSDig (options: measure, set scale) to measure a distance specified by the user (10 mm, in our example) on a ruler placed beside the leaf when it was scanned. The scale factor (mm/pixel) is used to convert coordinates from pixels to millimeters (or another unit of measure) and to have landmark configurations of all specimens to the same scale. If the scale factor is the same for all pictures, however, as in our case where all leaves were scanned using the same magnification and resolution, it can be set during the digitization of the first image only; this will be used for all other individuals in the same file.

We converted TPS into NTS (TPSUtil, convert TPS/NTS file option) checking the box for using the scale factor and also the one for using image names as labels. The NTS format is another ascii text file used for landmark coordinates (and other variables). The information on landmark coordinates stored in this type of file is the same as in the TPS format, but data are rearranged as a matrix with rows corresponding to specimens and columns corresponding to coordinates. In this example, the conversion to NTS is only used as a shortcut to quickly create specimen identifiers based on the original names of the image files.

Indeed, if descriptive names are chosen for the image files and used as labels in NTS, they can be easily converted into grouping variables in MorphoJ, after importing the NTS file (menu: “file”; option: “create new projet” using two-dimensional data in “NTSYSpc” format, without object symmetry as only one side was digitized). For instance, in a file name as Busso_T01_L1_R1.jpg or Campo_T04_L2_R2.jpg: the first five characters indicate the locality; the 7^th^ to 9^th^ a tree from that locality; the 11^th^ and 12^th^ a leaf from that tree with its first (R1) or second (R2) replica image on which landmarks were digitized once on each image. Using the option “extract new classifiers” in the menu “preliminaries” of MorphoJ, one can tell the program to use characters 1–5 for the geographic populations; 1–9 for the trees; 1–12 for the individual leaves. Classifiers, as well as covariates made of continuous variables (e.g., geographic coordinates, environmental covariates as temperature, humidity etc.), can also be imported later from separate txt files or specified manually in the edit classifiers menu of MorphoJ.

Finally, to aid visualizations, we drew lines, called links, between pairs of landmarks (menu: “preliminaries; option: create or edit wireframe”) to create a wireframe which resembles a stylized leaf. We also built and imported (menu: “file”; option: “import outline file”) a leaf outline to make a more effective graphical representation of the output of the analyses. Outlines are contours that are drawn in TPSDig using a series of points. These points will not be used in the analysis, as they are not landmarks, but can be used to show shape variation by rendering the contour image in the background. Outlines cannot be imported directly in the TPS format and they have to be converted in a ascii txt file format, as described in detail in MorphoJ user's guide.

After having completed data collection and preliminary operations, the numerical analysis begins:

Specimens are Procrustes superimposed (menu: preliminaries; option: Procrustes fit). In MorphoJ, as in most other GMM programs, this operation separates size and shape and also projects shape coordinates into a Euclidean space tangent to the Procrustes shape space.

The projection into the tangent space is performed because standard statistical methods such as regression, analysis of variance and many others generally require data to be in a flat Euclidean space. In simple terms, this means that a distance between two observations is a straight line computed using the theorem of Pythagoras (or its multivariate extension). However, because the Procrustes shape space is curved, it has to be approximated by a tangent Euclidean space using a projection computed as a cartographer would do to draw the curved surface of the Earth onto a flat map. The point of tangency between the two spaces is the sample mean shape. The approximation in the tangent space for almost all biological datasets analysed until now is excellent [5]. The space occupied by real organisms, even when it is a macroevolutionary study of differences between mammal orders [40], is tiny compared to the space of all possible shapes. The tangent space approximation is seen as a purely theoretical issue by the majority of morphometricians working on biological data. Nevertheless it should be checked. TPSSmall [37] regresses through the origin the set of Euclidean distances in the Euclidean space onto the set of Procrustes shape distances. If the approximation is excellent, one will get a regression with both slope and correlation virtually equal to 1.

The sample was inspected for outliers. This was done both on the total sample and within each population sample. Sub-samples are obtained in MorphoJ using the “preliminaries” option “subdivide dataset by” with an appropriate classifier. Outliers for size are easier to find using univariate methods as, for instance, box-plots [41] in PAST [42], [43]. For shape, MorphoJ has an option in the preliminaries menu that may help to detect specimens unusually distant from the mean. This is based on a model that assumes that the data are multivariate normally distributed. A second exploratory method to spot potential outliers consists in looking for individual points separated from the main scatter of observations in PCA scatterplots. A PCA can be performed in MorphoJ after computing the variance covariance matrix of the Procrustes shape coordinates (menu: “preliminaries”; option: “generate covariance matrix”) and projecting the data onto the corresponding eigenvectors (menu: “variation”; option: “principal component analysis”). A third option to aid outlier detection in combination with the previous two is to look for isolated branches, generally near the root of the tree, in phenograms. Phenograms can be computed by performing a cluster analysis in PAST [42], [43] using Euclidean distances calculated on the matrix of Procrustes shape coordinates (menu: multivar; option: cluster analysis). A phenogram is a summary of the similarity relationships in a multivariate dataset using a tree diagram. The distance among specimens in the tree is proportional to their differences. The most similar shapes are on sister branches, the most dissimilar ones are isolated next to the root. Trees tend to distort shape distances [44]. The index of cophenetic correlation available in PAST helps to quantify the magnitude of the distortion [45]. The index is computed as the correlation between the original shape distances and the distances reconstruced using the topology of the tree and ranges between 1 (no distortion) and 0 (maximum possible distortion). Different tree building algorithms may be used and the magnitude of their cophenetic indexes compared to select the one which minimizes ovarall distortions.

#### 2a) Testing variation in populations, trees and leaves using a modified Procrustes ANOVA: theoretical background

For clarity, when *populations*, *trees*, *leaves* and *replicas* (i.e., *error*) refer to effects being statistically tested, we will be now using italics. Differences in leaf size and shape can occur at several levels. Our main purpose is to measure and test variation in geographic populations. We also need to know, however, how much leaves differ within and among trees and whether this is more than explained by measurement error. For this aim, we used a hierarchical analysis of variance (ANOVA) with *populations* as the main effect, *trees* and *leaves* as random effects (i.e., factors whose number of levels is not set by any objective a priori criterion and just reflect sampling), and *leaves* nested in *trees*. In the “variation” menu of MorphoJ, this analysis is called “Procrustes ANOVA” after Klingenberg et al. [25]. Variance is partitioned by using a “hierarchical sum of squares” (p. 620, [41]) in a way such that each effect is adjusted for all other effects that appear earlier in the hierarchy. This is taking into account the nested structure of the data (an issue that is crucial if the design is unbalanced, i.e., with unequal sample sizes), thus allowing one to quantify differences in *populations*, *trees* regardless of population and *leaves* regardless of both population and tree. The variance unexplained by any of these effects is measurement error and it is estimated using the differences between repetitions, which include both digitizing error and the error during image acquisition. Thus, in summary, we decomposed total variance in size or shape into main (*populations*), random (*trees*, *leaves*) and error (*replicas*) components and computed ratios between these components (*populations*/*trees*, *trees*/*leaves*, *leaves*/*error*) corrected by the appropriate number of degrees of freedom to generate the test statistics.

#### 2b) Testing variation in populations, trees and leaves using a modified Procrustes ANOVA: software applications

The Procrustes ANOVA in the variation menu of MorphoJ was designed for studies of asymmetry in bilateral symmetric structures [25]. The analysis is automatically performed for both size (univariate) and shape (multivariate). It is crucial that the factors are accurately specified when the analysis is requested, because it is a hierarchical model and therefore the order of the effects (first *populations*, then *trees*, followed by individual *leaves*) is important. The current version of MorphoJ does not allow one to specify random effects other than individuals (i.e., *leaves* in our study). Thus, we had to take a few additional steps to obtain the correct result:

We selected *populations* followed by *trees* as main effects (right panel in the analysis window) and *leaves* as the only random effect (first scroll down in the menu in the left side of the analysis window). This is a misspecification of factors and determines that both *populations* and *trees* are incorrectly considered at the same level with each of them being compared to (i.e., divided by) the individual *leaves* mean sum of squares.

We manually computed the F ratio for the main effect of *populations* using *trees* as a random effect to correct for the misspecified model. The computation consists in dividing the *populations* mean sum of squares by the *trees* mean sum of squares and it is straightforward because the mean sum of squares and the corresponding degrees of freedom are the same as in the standard output of MorphoJ. Thus, one simply needs to take the numbers from the result window of MorphoJ and manually do the ‘*populations* to *trees*’ F ratio.

Finally, it is possible to obtain the significance P level of the observed F statistics using an F distribution calculator (e.g., http://davidmlane.com/hyperstat/F_table.html. Accessed 2011 June 8) or a table of critical values (http://www.itl.nist.gov/div898/handbook/eda/section3/eda3673.htm. Accessed 2011 June 8) with the degrees of freedom corresponding to *populations* (numerator) and *trees* (denominator).

To complete the analysis, we also calculated the percentage of sum of squares explained by each effect. The sum of squares measures the deviation of the observations from the mean (or means, when groups are present) and is more accurately referred to as ‘sum of squared deviations’. As it is an estimate of the variability in the sample which is readily available in the ANOVA output, it can be used to assess how well different factors fit the data. This is easily obtained by dividing the sum of squares of an effect by the total sum of squares and multiplying this ratio by 100. However, this is not the same as estimating variance components in the ANOVA, a more advanced statistical procedure that we briefly explain in a note in the Appendix 1.

#### 3) Testing population differences using permutation tests and discriminant analyses: theoretical background and software applications

Results of the Procrustes ANOVA provide a basis on which to plan the next steps of the analysis. The lowest level, *leaves*, must be statistically significant, as differences among leaves regardless of *populations* and *trees* must be larger than measurement error. Measurement error should, therefore, explain a negligible percentage of variance. The next level, *trees*, indicates whether there is more variation in leaves of different trees than within the same tree. If that is the case, there is a stronger justification to pool leaves within trees and use their averages as an estimate of the trend in leaf form in each tree. Having removed pseudo-replicates (i.e., non-independent observations as multiple leaves from a tree) from samples, a variety of standard tests for group differences can be applied [12], [13], [46] to examine the highest and most interesting level, at least from a taxonomic perspective, of group variation: population differences. Thus:

First we averaged leaves within trees in MorphoJ using the option “average observation by” from the menu “preliminaries”. Then, we used the averaged data for testing *populations* using a series of tests for sample mean differences including an estimate of the accuracy of leaf shape in predicting groups.

For size, we performed a parametric t-test for independent samples. We also repeated the test using permutations, which do not assume normally distributed data and can be performed even if samples are small. All these tests are simultaneously performed in PAST using the menu “Statistics” with the option “F and T test for two samples”. Permutation tests for group differences can also be done in MorphoJ using a regression approach. A dummy covariate is created (menu: preliminaries; option: edit covariates), where one population is coded as -1 and the other as 1 (or vice versa). Then, size is regressed onto this dummy covariate using permutations to test significance. This test provides a P value together with the percentage of variance explained by *populations*. In this and other cases when we express the fit of the model in terms of variance instead of sum of squares, we do so because variance is a concept most readers may feel more familiar with. As there is a single set of predictors and no partitioning among factors, as in the Procrustes ANOVA, the percentage of sum of squares explained is identical to the percentage of variance (Zelditch et al., 2004).

Multivariate shape differences can be tested pairwise in PAST using a parametric approach (menu: Multivar; option: Hotelling) or permutations (menu: Multivar; option: Two-group permutation). For those unfamilar with methods using randomizations, the rationale for permutation tests and their multivariate extension are well explained in the chapter on “Computer-based statistical methods” in Zelditch et al. [26] and also in the introductory book on resampling statistics by Manly [47]. An important caveat to bear in mind using these tests is that, although permutations can be performed with sample sizes too small for parametric tests, small samples will inevitably reduce statistical power and increase inaccuracies in estimating group means and variances. As for size, differences can also be tested in MorphoJ using the regression approach and permutations. Tests of mean sample differences are obtained in MorphoJ also as part of the output of a discriminant analysis (DA). Results will be equivalent to those using the regression approach but with the latter one also computes the variance explained and with the former one also produces a classification table, as explained in the next paragraph.

We tested group differences also using a DA. In MorphoJ this is obtained from the menu “comparison”, option “discriminant analysis”. The DA is probably the most widely used statistical method for investigating taxonomic differences and is generally used as a synonym for canonical variate analysis (CVA). The term DA is preferentially used when only two groups are compared; CVA when there are three or more groups. Often in a DA on taxonomic data the main focus is on group prediction, whereas that in a CVA is more on ordinations. Neff and Marcus [35] and Albrecht [48] provide excellent and concise introductions to DA/CVAs; Klingenberg and Monteiro [49] discuss its use in GMM. In simple terms, a DA/CVA is another method to combine a set of variables, as the PCA. However, the linear combinations of the original variables are now derived to maximize group separation for: 1) testing groups (statistical inference), 2) plotting their differences (ordination) and 3) predicting group affiliation (classification). All three types of output are produced by MorphoJ when the analysis is specified as DA for two groups (as in our case). However, with three or more groups, the CVA option in MorphoJ will only test group differences pairwise and produce ordinations. The classification table and the test for overall (i.e., all groups together) differences can be obtained using PAST (menu: “Multivar”; option: “MANOVA/CVA, confusion matrix”). Thus, Procrustes shape coordinates can be exported from MorphoJ as a text file (menu: “file”; option: “export dataset”) and directly opened in PAST after changing the name of the first column from ID to LABELS (or anything else than ID). Alternatively, raw coordinates in TPS format could be directly imported in PAST, GPA superimposed, subjected to a PCA and then used for a CVA. In PAST, groups are specified by selecting and colouring rows (menu: “Edit”; option: “Row colour/symbol”) and multivariate tests should be performed using PCs with non-zero eigenvalues to be sure that degrees of freedom are correctly computed from Procrustes shape data. Finally, both in MorphoJ and PAST, only jack-knife cross-validated classification tables provide reliable information on groups [50]. In the jack-knife or leave-one-out cross-validation, one by one each individual is left out from the analysis and predicted using data from all other specimens. This way the jacknifed predictions avoid the ‘circular reasoning’ and consequently inflated accuracy of a non-cross-validated DA/CVA, where a specimen is classified using functions that were calculated on samples that included that same specimen. Both non-cross-validated and cross-validated results are produced in MorphoJ, whereas cross-validated results must be requested in PAST by checking the appropriate box in the confusion matrix window.

#### 4) ‘Size-correction’ after testing the effect of size on shape (i.e., allometry) using a multivariate analysis of covariance (MANCOVA) design: theoretical background and software applications

Size and shape have, up to this point, been tested separately. It might be interesting to consider also the way they may interact and covary. For instance, if size variation is large, one may want to repeat shape comparisons after controlling for the effect of size on shape [51]. This effect is called allometry and in general terms refer to a change in shape associated with size differences [52]. Allometry can account for a large and statistically significant proportion of morphological variation. This is tested using a multivariate regression of shape onto size (in MorphoJ, menu: Covariation; option: Regression). Centroid size may be first transformed to its natural logarithm to increase the fit of the model, which is estimated by the percentage of shape variance explained by size. Significance is tested using a parametric test (PAST) or permutations (MorphoJ).

If groups are present, one cannot fit a single regression line through all groups to test allometry. This is because lines could have group-specific slopes or intercepts. The standard parametric test for differences in slopes and intercepts of allometric trajectories also provides a method to ‘correct’ for the effect of size on shape. This is a MANCOVA with *populations* as groups and centroid size as a covariate [26]. A MANCOVA is similar to a MANOVA, in that one has a priori groups to compare, but also to a regression, as one can include one or more continuous variables as predictors. The aim is to test groups (*populations*) after removing the variance in the response variables (shape) accounted for by the covariate (size). By doing this, one may be able to say if differences in shape are actually the result of size variation only. Controlling for one factor, while testing for another one, makes simpler explanatory models and increases statistical power.

The MANCOVA was applied to averaged tree leaves we have already tested for *populations* differences in step (3). It was also used to compute ‘size-corrected’ shapes, which were then examined using the same series of tests as on the full shapes (3):

Before proceeding with the MANCOVA, the significance of allometry within groups could first be tested. This requires splitting populations into separate samples (MorphoJ menu: “Preliminaries”; option: “Subdivide dataset by”) and performing multivariate regressions of shape onto size one group at a time (menu: “Covariation”; option: “Regression”). If at least one of the groups is statistically significant, controlling for allometry using the MANCOVA, as described in the next paragraphs, might be interesting.

A full MANCOVA with *populations* as groups, size as covariate and the *populations* by size interaction term included is performed. The main aim is to compare regression slopes between groups. These are tested by the interaction between *populations* and size. In this context, here and throughout the rest of the paper, we informally use ‘interaction’ as a concise way to indicate the test for slopes using the same convention as in most statistical programs, although this does not rigorously correspond to the meaning of ‘interaction’ in a MANOVA. The test for slopes compares the amount of variance explained by two models: one is simultaneously fitting group-specific multivariate linear regressions with each population having its own slope; the second one is also fitting group-specific lines but it does so by forcing them to be parallel. The fit of the first model (i.e., the percentage of variance explained) will always be better than that of the second one, as to keep parallel lines regression slopes become a compromise between group-specific slopes. However, if separate lines fit the data only slightly better than parallel ones and this is not enough to be statistical significant, differences between the two models are negligible and allometric trajectories can be considered parallel. In terms of shape variation, this means that the allometric pattern is the same across groups: as the leaf becomes bigger, the relative changes in proportions of its regions are similar in the different *populations.* For instance, population A could have an obovate leaf whereas population B might be ovate but in both A and B bigger leaves will tend to be slender compared to smaller ones. The shape of the leaf is not the same, but the trend of covariation with size is the similar.

When slopes are different, allometric trajectories are pointing to different directions and one cannot easily control for the effect of size on shape in tests of group differences [26]. However, if (and generally only if) slopes do not differ significantly (i.e., the size by population interaction term of the MANCOVA is not statistically significant), one can proceed to the next step: controlling for the effect of allometry while testing groups and computing ‘size-corrected’ shapes. The MANCOVA is repeated after removing the (non-significant!) interaction term. The grouping variable, *populations*, is now testing differences in regression intercepts by comparing the percentages of variance explained by different models. However, the best-fit model in this instance is the one with separate but parallel lines, we have already seen, and the lower fit model is a single regression line through all data regardless of groups. If the difference is not statistically significant, intercepts of the two (or more) parallel lines with the Y axis are statistically indistinguishable. That means that allometric trajectories overlap among groups and therefore patterns are not only similar (i.e., parallel or laterally transposed) but actually the same. Group differences in this specific case are fully explained by allometry: if a population is bigger and shape covaries with size, its shape will be different simply because it has gone a little bit further along the same allometric trajectory.

To reconstruct ‘size-corrected’ shapes, slopes must not be statistically significant. The ‘size-correction’ according to the MANCOVA model can be performed in MorphoJ. However, MorphoJ does not test slopes and assumes that this has already been done in another software. For instance, one can use TPSRegr [37] to perform a MANCOVA of two dimensional shapes following a regression approach. A detailed description of the analytical steps involved in this test is available in the help file of TPSRegr. Having demonstrated the non-significance of slopes, one can proceed with the ‘size-correction’ in MorphoJ. This is specified in the regression window (menu “Covariation”) using shape as the dependent dataset, centroid size as the independent one and the option “pooled regression within subgroups” with *populations* as subgroups. MorphoJ then performs a series of operations:

- fits parallel regressions lines;
- takes a straight line perpendicular to the size X axis;
- finds the points of intersection between this (b) line and the regression lines of each population (nota bene: the intersection is a point in the multivariate shape space, which has as many dimensions as the number of shape coordinates, i.e. twice the number of landmarks in two-dimensional data);
- computes the regression residuals, that is the differences between each observed shape and its prediction according to the population-specific parallel allometric trajectory;
- adds population by population (c) to (d) to reconstruct shapes in which the within-group allometric variation has been removed.

The result is a sample of ‘size-corrected’ shapes according to group-specific parallel allometric trajectories. They can be used for further analyses (e.g., DA and permutation tests as in step (3)) by selecting the output of the regression in the project tree window of MorphoJ. Although MorphoJ refers to the ‘size-corrected’ shapes simply as “residuals”, they actually are residuals added to shapes computed as in (b–c). Because the (b–c) shapes are predicted at the same common size within each group and the residuals (d) are by definition independent on size, adding them back to (b–c) creates new samples whose means (b–c) are ‘size-corrected’ (d–e) and where allometric variation has been ‘squeezed’ around the (b–c) means. Thus, by using a common size to predict shapes we made the effect of size on shape comparable among groups. However, we have not said what this common size (b) might be. It turns out that one can use the grand sample mean size (i.e., the mean size of all groups) or any other size and in terms of statistical testing and relative differences among groups it will not make any difference. This is because regression lines are parallel and therefore their relative distances (i.e., the shape differences) are the same across the whole range of size variation. If this assumption is violated, because slopes appreciably different, results of the ‘size-correction’ could be different depending on the choice of the common (b) size [26].

#### 5) Interpretation and visualization of shape variation: theoretical background and software applications

This last section explains how shape variation is interpreted and visualized after statistical analyses. In traditional multivariate morphometrics the interpretation of results largely relies on the determination of the relative importance of the different measurements. For instance, in a PCA, it might be interesting to know which variable contributes most to the main axis of variation. In a regression context, the association between predictor(s) and predicted variables might be stronger or weaker depending on the variable. This type of interpretation is mostly done by looking at the coefficients (PC loadings, regression coefficients etc.) used to weight the variables. In GMM this cannot be based on coefficients and must done using diagrams to visualize shape differences between two groups, variation along a vector (PC1, PC2 ... or a regression vector etc.).

Several types of diagrams are available in MorphoJ, PAST and other programs:

Thin plate spline (TPS) deformation grids [26], [53] are one of the most effective and commonly used shape diagrams. They take their inspiration from D'Arcy Thompson transformation grids [54]. Thompson's idea was to describe shape changes by superimposing a rectilinear grid onto a starting form, for instance a fish, and then use simple mathematical transformations to warp this grid in order to match a target, a fish with a different shape. Similarly a “...way to think about … [the] TPS ... is as if one form were printed on a transparent stiff plastic sheet [together with a set of square grids] and then manipulated by bending so that its ‘shadow’ takes on the prescribed landmark positions of the second form” [55]. In practice, what the TPS does is using an interpolating function to produce smooth deformations. The smoothing is done by minimizing the curvature of the sheet where the landmarks in the starting form are placed and manipulated until they overlap with those in the target configuration. This operation produces vectors of coefficients which can be used to predict how grid lines may change because of the warping.

Rendering the contours (often called outlines) of a study structure like a leaf or even a picture or a three-dimensional surface on which landmarks were digitized is another option to visualize shape variation. This is generally achieved by applying the same coefficients obtained by using the TPS to predict shape changes in the outline or any other type of visual information ‘drawn’ in the space of the landmark configuration. Although there is not quantitative information in these diagrammatic representations except where the landmarks are, they help to interpret shape differences and make them more tangible than the abstract visualization of landmarks alone.

The relative differences between two specimens can be visualized using displacement vectors. Displacement vectors (called “lollipop graph” in MorphoJ) are arrows drawn between a landmark in a starting shape and the same landmark in a target shape. They can be useful and effective, as long as one carefully integrates the information provided by each of these arrows over the whole landmark configuration. Otherwise, they may give the misleading impression of a landmark moving independently from one location to another. Landmarks do not move. It is the space which they define that changes (expands, contracts, bends etc.). Differences between two shapes, for instance, an equilateral and an isosceles triangle, is the same regardless of how we superimposed them to extract size and shape variables. However, when shown one superimposed on top of the other, the relative position of landmarks becomes a function of the type of superimposition and Procrustes is the standard choice only for its statistical properties. In the Discussion we will consider one of several other types of superimposition and show it is likely to be equivalent in terms of numerical outputs in multivariate analyses, but may suggest very different shape differences, if these are described using superimposed shapes and displacement vectors.

Finally, a fourth and most common type of visualization is the one using wireframes, which we have already described in the first section of the methods.

All these types of visualization are readily available for two dimensional data in MorphoJ, which also does three dimensional wireframes and displacement vectors. By right clicking in the graphics shape changes window and selecting “change type of graph” one can choose the diagram. In PAST some of the analyses (e.g., a PCA of shape coordinates) allows one to make visualizations using wireframes and TPS grids. PAST also implements a system of colour coding called Jacobian expansion factors to help detecting using different colours regions where the grids are shrinking from those where they are expanding.

A final consideration concerns the magnification of differences. It is customary to magnify small variation to aid its interpretation. This is because small but significant differences can be difficult to see unless they are ‘exaggerated’. The magnification consists of a simple scalar multiplication of shape differences between a starting shape and its target. For instance, if an observation has a score on PC1 of 0.05, a two-fold magnification of the shape differences between this observation and the mean shape is the shape that one would get by doubling the lengths of the displacement vectors and it is the shape whose score on PC1 would be 0.1. As this is not a real shape, one needs to be explicit about the magnification factor, whenever possible consistently use the same magnification in all diagrams, and be careful to avoid the overinterpretation of tiny amounts of variation.

## Results

### 1) From landmark configurations to shape variables

The tangent space approximation was tested in TPSSmall [37]. The slope of the regression of Euclidean distances in the tangent space onto Procrustes shape distances in the curved Procrustes shape space was 0.997 with a correlation of 1.000. The mean and maximum Procrustes shape distances to the sample mean shape were 0.110 and 0.252 units of Procrustes shape distance. The approximation was therefore excellent.

**Figure 2:**
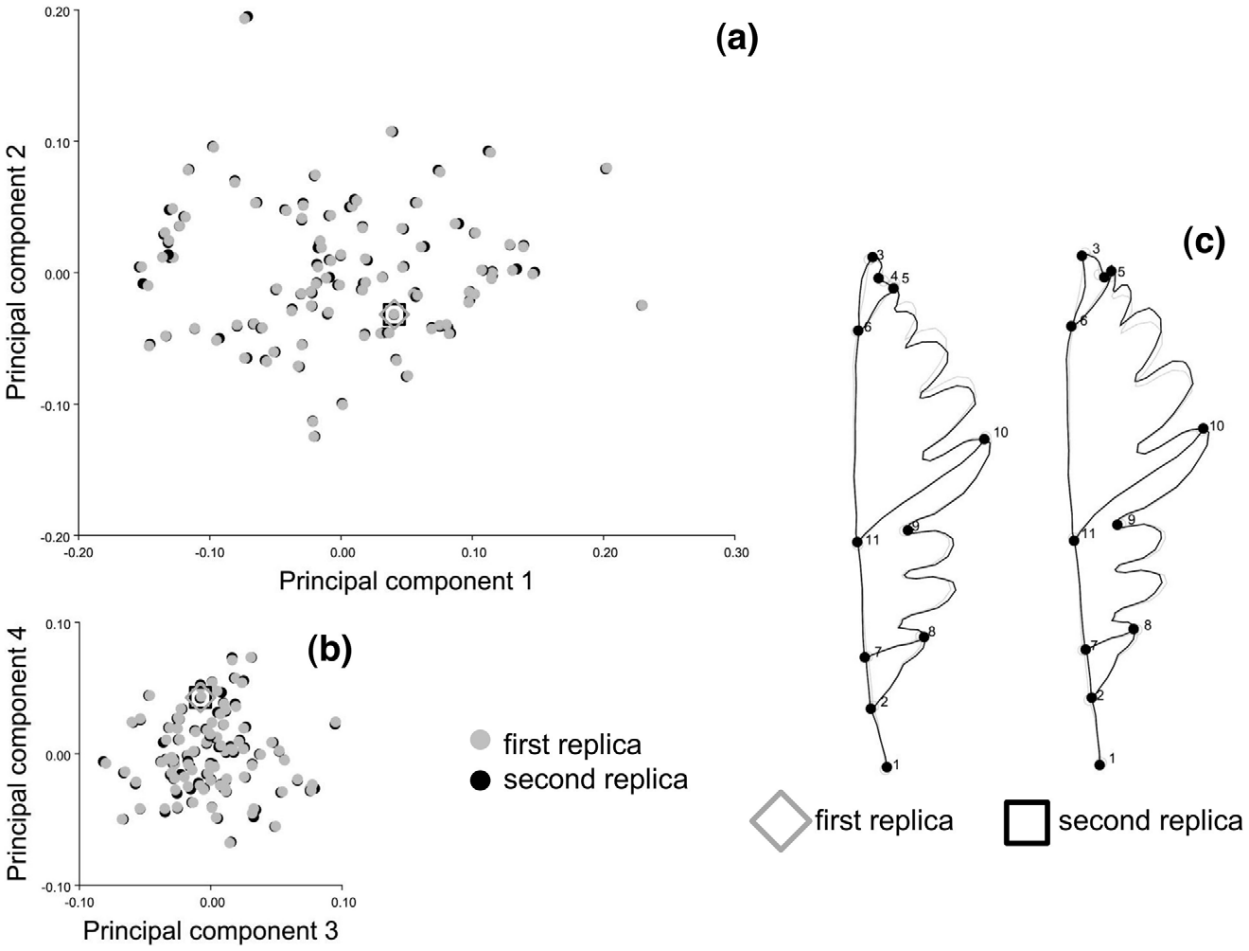
Shape variation including all observations and replicas. Scatterplots of PC1 *vs* PC2 (a), and PC3 *vs* PC4 (b), which overall explain 88.3% of variance. As an example, the first and second replicas of the specimen ‘Campobasso 14a’ are visualized using outline drawings magnified 10 times (c); full shapes are shown in the visualization and square and diamond symbols are used to indicate the position of the replicas of this specimen in the shape sub-spaces of the first PCs.

Raw coordinates were imported in MorphoJ, Procrustes superimposed and subjected to a PCA. A preliminary screening for outliers (results not shown) showed a good correspondence between the observed data and shape distances expected under a multivariate normal distribution model, no long tails in the distribution and no evident outliers. Also, scatterplots of several of the first PCs of shape (Figure 2 shows pairwise plots of PC1 vs PC2 and PC3 vs PC4) and a phenogram built using the unweighted pair group method with arithmetic mean on the matrix of Procrustes shape distances (not shown) suggested that there were no specimens with unusual shapes. Box-plots and histograms of size (not shown) also did not suggest the presence of strong outliers, although the distribution of Campobasso leaves was skewed.

### 2) Testing variation in populations, trees and leaves using a modified Procrustes ANOVA

A modified Procrustes ANOVA was performed on all specimens (including replicas and multiple leaves from each tree). The analysis had to be specified in MorphoJ using both *populations* and *trees* as main effects and *leaves* as the individual random effect. As anticipated, this is not the correct design, but the current version of the program does not permit a second random effect in the model. *Trees* was made into a random effect by manually computing the appropriate F ratio using mean sum of squares and degrees of freedom from the original output of MorphoJ, as described in the Materials and Methods. Thus, we corrected the F ratio for the *populations* term so that this effect (numerator) could be compared to the variation among *trees* (denominator) instead of that between *leaves*, as in the original output.

Results for size and shape are reported respectively in Tables 2–3. For size, the main effect of populations was statistically significant and explained about 6% of total sum of squares. Differences in leaf size among trees were only slightly but non-significantly larger than differences between leaves of the same tree (respectively ca. 55% and 39% of total sum of squares). The individual effect (i.e., *leaves*) was highly statistically significant and measurement error accounted for less than 0.1% of total sum of squares. This meant that, for size, variation in leaves had a fit to the data which was two orders of magnitude larger than the error in image acquisition and landmark digitization.

**Table 2:** Centroid size variation.

effect	explained SS	SS	MS	df	F	P
*populations*	6.2%	55.578	55.578	1	4.862	0.033
error for locality		491.517	11.431	43		
*trees*	54.7%	491.517	11.431	43	1.400	0.137
error for tree		350.970	8.162	43		
*leaves* (individual)	39.1%	350.970	8.162	43	41971.891	<0.0001
error for individual^1^	<0.1%	0.017	0.000	88		
total	100.0%	898.083				

Hierarchical sum of squares ANOVA: main effect: *populations*; random factors: *trees*, *leaves.* Here and in the following tables, SS, MS and df refer respectively to sum of squares, mean sum of squares (i.e., SS divided by df) and degrees of freedom.

1 Measurement error.

**Table 3:** Shape variation.

effect	explained SS	SS	MS	df	F	P
*populations*	2.8%	0.066	0.003686	18	1.916	0.012
error for locality		1.489	0.001924	774		
*trees*	63.0%	1.489	0.001924	774	1.846	<0.0001
error for tree		0.807	0.001042	774		
*leaves* (individual)	34.1%	0.807	0.001042	774	1340.927	<0.0001
error for individual^1^	0.1%	0.001	0.000001	1584		
total	100.0%	2.364				

Hierarchical sum of squares Procrustes ANOVA: main effect, *populations*; random factors: *trees*, *leaves*.

1 Measurement error.

Results for shape largely mirrored those for size with a single main exception: the random effect of *trees* was highly statistically significant and explained almost twice the sum of squares explained by individual leaf variation. Thus, for shape, population differences are small but statistically significant, tree differences are appreciably larger than variation in leaf shape within trees and measurement error is completely negligible. Figure 2 shows an almost perfect overlap between the two replicas of each specimen on scatterplots of the first four PCs. The same result holds up to the 6–7^th^ PC, which cumulatively account for about 95% of variance (results not shown). Differences between replicas are so small that in the example specimen of Figure 2 they can be hardly detected after a 10 folds magnification.

### 3) Testing population differences using permutation tests and discriminant analyses

Leaves from the same tree were generally more similar than leaves of different trees (see previous section). They were averaged within *trees* and the averaged data used to further examine population differences. They were subjected to a second series of parametric and permutation tests for group mean differences and the group predictive accuracy of shape was tested in a DA.

**Figure 3:**
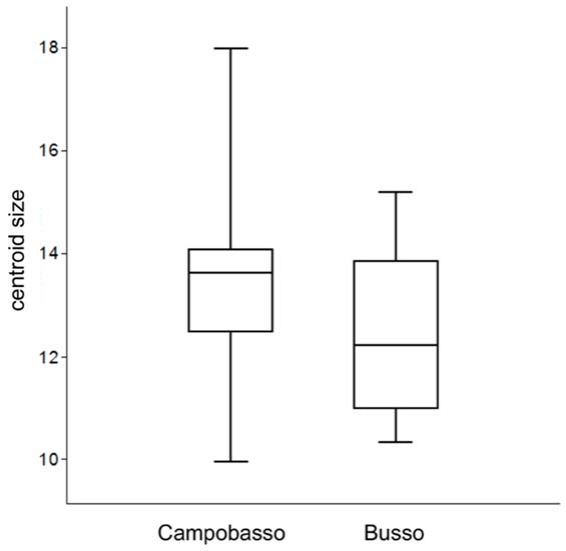
Size variation after averaging leaves within trees. Box plots (drawn in PAST): median, 25–75% quartiles, minimum and maximum.

**Figure 4:**
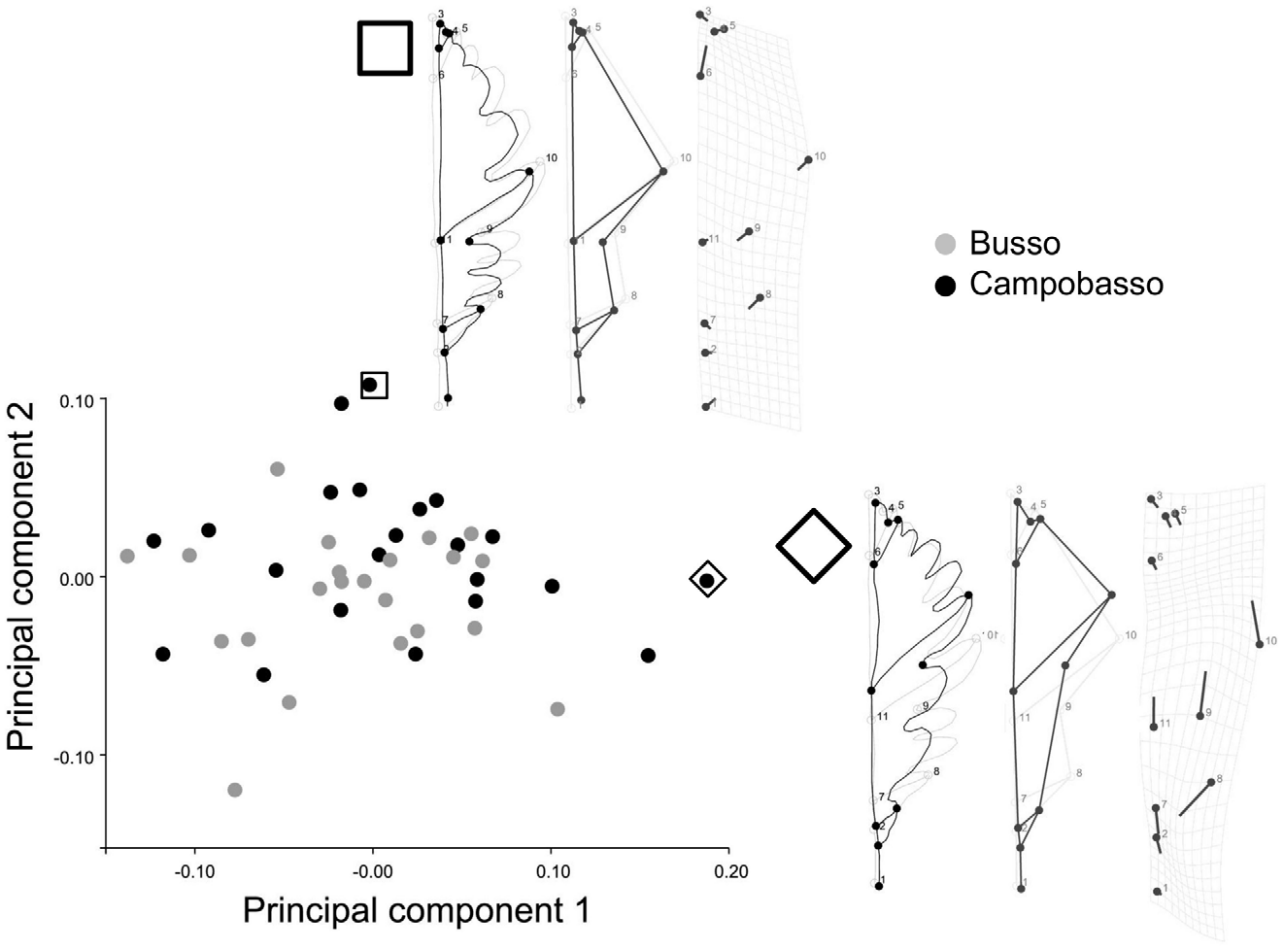
Shape variation after averaging leaves within trees. Scatterplots of PC1 *vs* PC2, which together explain 74.2% of variance. Shapes are visualized for the positive extremes of these axes using outline drawings; there is no magnification and square/diamond symbols are used show the positions of visualized shapes in the PCA scatterplot.

**Figure 5:**
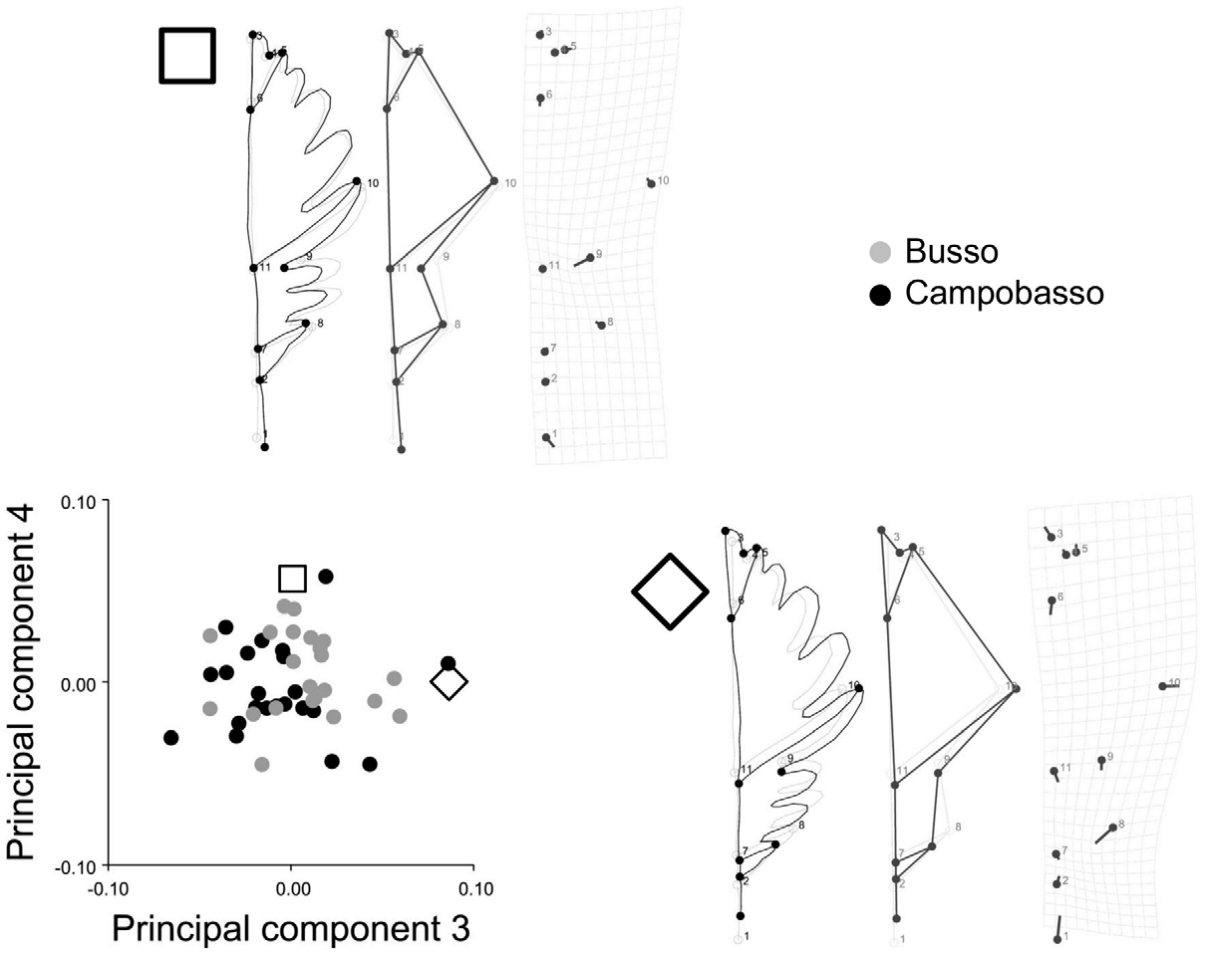
Shape variation after averaging leaves within trees. Scatterplots of PC3vs PC4, which together with the first two PCs (Fig. 3) explain 90.3% of variance. Shape are visualized using the same conventions as in Figure 4.

Results of significance tests are shown in Table 4. Different methods produced congruent results. Size was statistically significant (P<0.05), as in the Procrustes ANOVA, and population differences explained 10.2% of variance. The box-plot in Figure 3 shows that Campobasso leaves tend to be slightly larger than Busso, but there is a lot of overlap between the two populations. Shape in contrast was not statistically significant and *populations* only explained 4.3% of total variance. The pattern of variation is summarized in Figures 4 and 5 with scatterplots for the first four PCs of shape. The two populations mostly overlap. Shape diagrams (leaf outlines, wireframes and TPS grids) for the positive extremes of the PCs suggest a large amount of variation in the samples. Positive extremes of PC1, PC3 and PC4 are characterized by either an enlargement or an elongation of the distal half of the leaf relative to the proximal half. PC2, in contrast, indicates narrowing of the leaf along its entire length. On PC1, PC3 and PC4 differences mostly concerned very localized shape changes. In some more details, PC1 is related to changes in the elongation of the leaf blade from sub-elliptical (negative extreme) to obovate (positive extreme) and in the leaf base from cordate (negative extreme) to acute (positive extreme). Also, the largest width of the leaf blade seems to be pushed distally towards to the apex of the leaf by the elongation of the base and vertical compression of the blade (postive extreme of PC1). The shape variation accounted for by PC2 is principally related to the constriction of the apical region and to the narrowing of the width of the leaf. The positive extreme of PC3 shows a leaf with a shorter petiole, a constricted basal region, an enlarged leaf blade and a more acute apical region; this trend is reversed towards the negative extreme. Finally, PC4 indicates differences in the deepness of the lobes, which is remarkably pronounced at the positive extreme.

**Table 4:** Differences between means of *populations* after averaging leaves within trees.

SIZE	t-test	df	P		P(perm.)		
	2.179	1, 42	0.035		0.0350		
SHAPE	T-square	df	P	Mahalanobis d.	P(perm.)	Procrustes d.	P(perm.)
total	44.942	18, 25	0.177	2.021	0.1780	0.0388	0.1336
‘size-corr.’	51.211	18, 25	0.110	2.158	0.1076	0.0315	0.2474

Permutation tests with 10000 random permutations.

**Figure 6:**
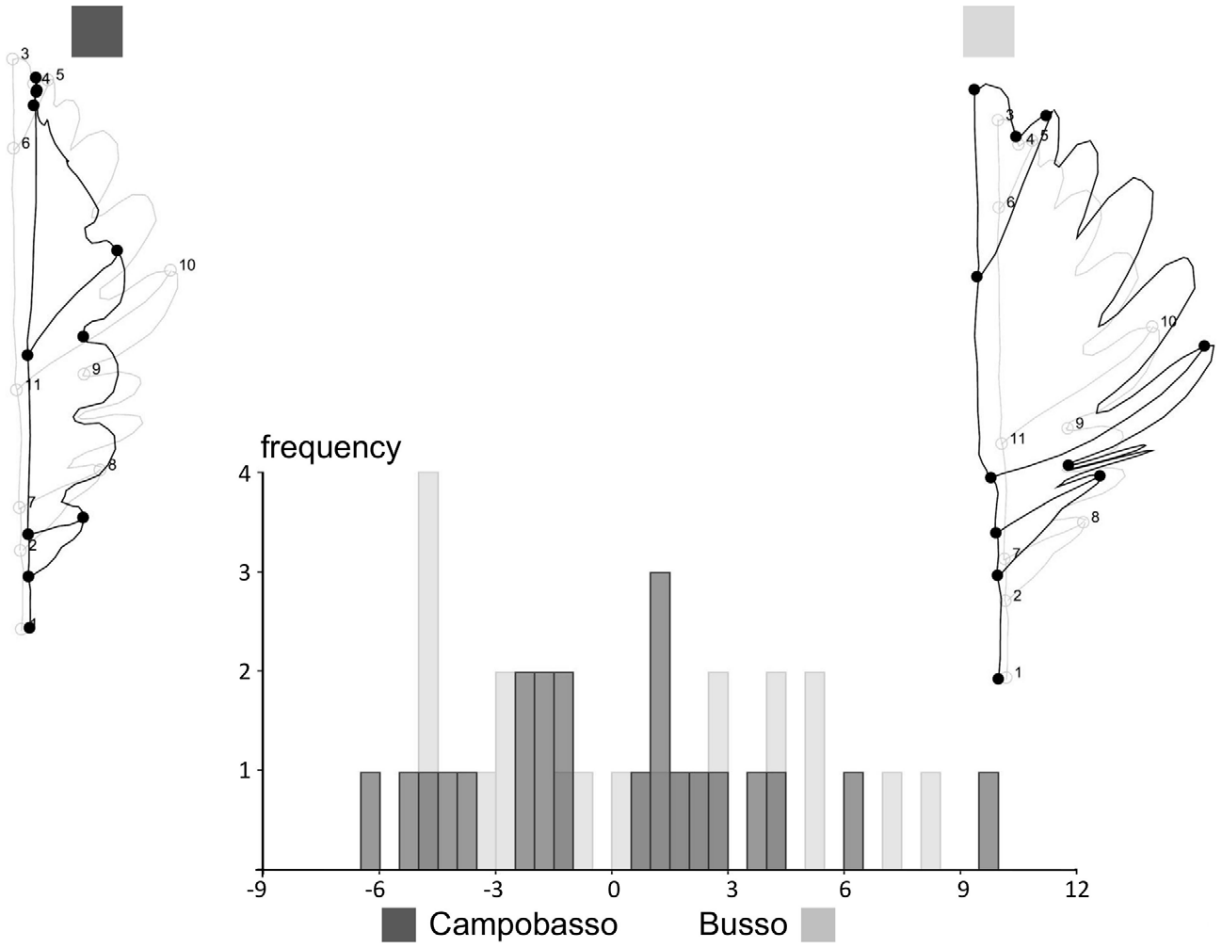
Discriminant analysis of geographic populations using leaf shape after averaging within trees. Frequencies of discriminant scores predicted by a jacknife (leave-one-out) cross-validation are shown using histogram bars; population mean shapes are visualized using outline drawings magnified 10 times.

As in the PCA, the results of the DA demonstrate that the range of leaf shapes largely overlapped between the two populations (Figure 6). As there are only two groups, there is a single axis of shape differences and scores are shown with histogram bars proportional to their frequency. The mean leaf of Campobasso is somewhat slender compared to the wider one of Busso. However, these differences had to be magnified 10 times to make them visible. Since they do not reach significance, they should be interpreted with the greatest caution and will have to be confirmed on larger samples. The cross-validated classification table (Table 5) shows that the accuracy of leaf shape in predicting populations is hardly better than a 50% random chance.

**Table 5:** Differences between *populations* after averaging leaves within trees.

all shape	Campobasso	Busso	‘size-corr.’	Campobasso	Busso
Campobasso	50.0%	50.0%	Campobasso	59.1%	40.9%
Busso	40.9%	59.1%	Busso	36.4%	63.6%

Jacknife cross-validated classification table using all shape or only ‘size-corrected’ shape in DAs.

### 4) ‘Size-correction’ after testing the effect of size on shape (i.e., allometry) using a MANCOVA design

**Figure 7:**
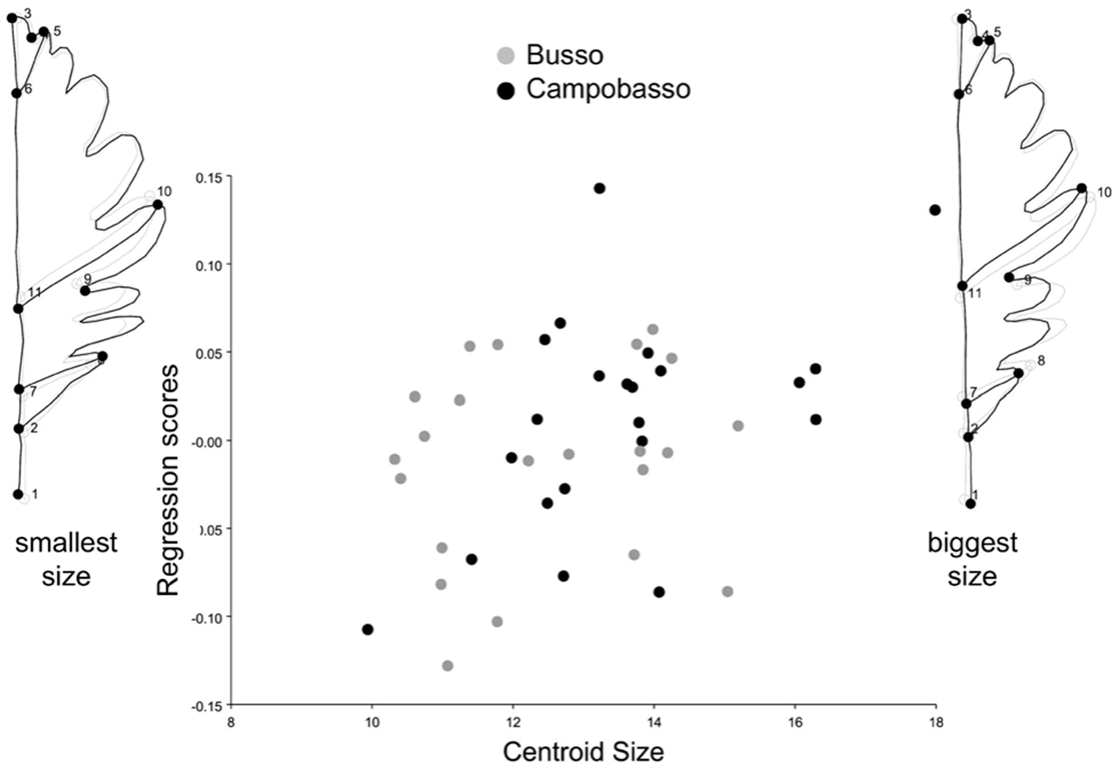
Regression of shape onto size pooling within *populations*. Scatterplot of regression scores (i.e., the projection of shapes in the direction of the vector of regression coefficients, Drake and Klingenberg, 2008) *vs* centroid size; shapes at the opposite extremes of the range of allometric variation are shown using leaf outlines with no magnification.

**Figure 8:**
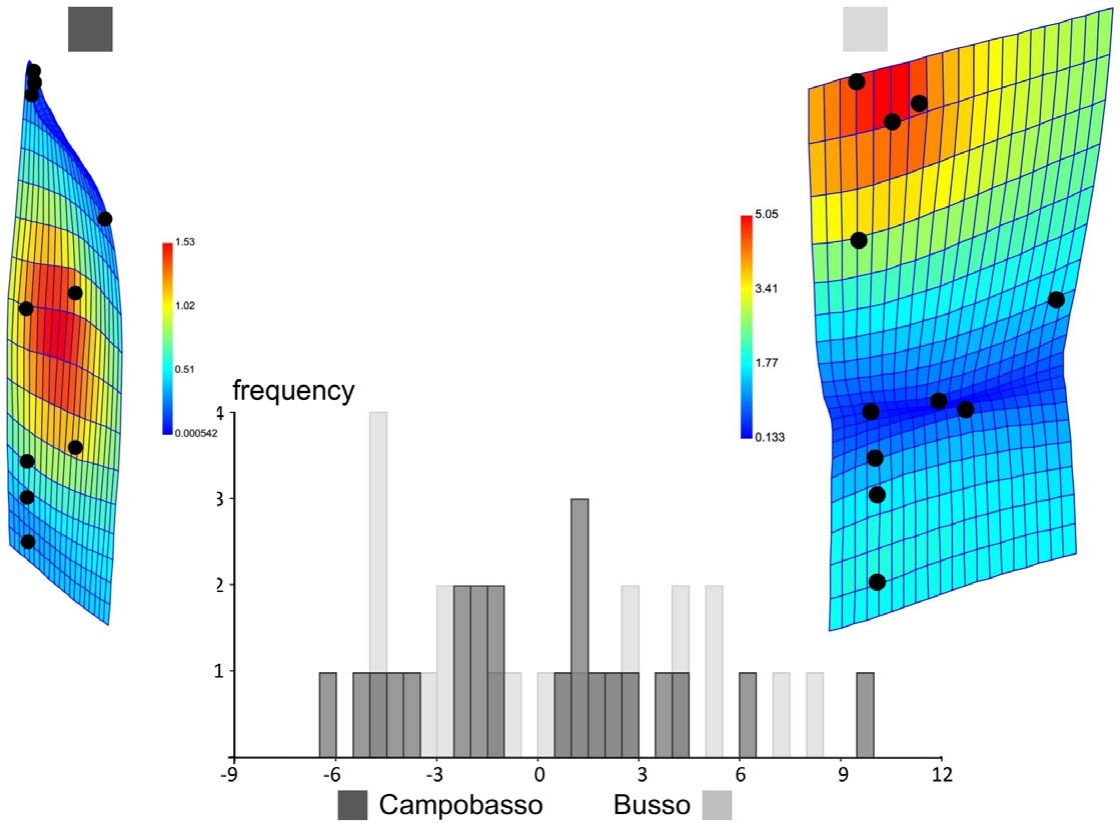
Discriminant analysis of geographic populations using leaf shape after averaging within trees. Same differences as in Figure 6 (printed black and white version) visualized in PAST using TPS deformation grids and colour coded Jacobian expansion factors which measure the degree of local expansion or contraction of the grid: yellow to orange red for factors >1, indicating expansions; light to dark blue for factors between 0 and 1, indicating contractions).

Centroid size was not log-transformed, as the transformation did not appreciably improve the fit of the model and made no difference in the results (not shown). Regressions of shape onto size one population at a time (not shown) were marginally statistically significant (P = 0.06, 11.4% of variance explained) only for Campobasso. This is indicative of a modest and probably negligible allometry. ‘Size-corrected’ data are therefore unlikely to produce results different from the analysis of full shape. One may still want to perform the analysis to be confident that this is indeed the case. In this study we run the MANCOVA mainly to provide an example of this model. Results are shown in Table 6. The fit of the different MANCOVA models measured by the percentage of variance explained varied from 8% (separate lines for populations) to 4% (single line regardless of population). The interaction term (test for slopes) was not statistically significant. Therefore, it was removed and the MANCOVA was repeated. Also *populations* (test for intercepts) were not statistically significant. This test as well as the large overlap between populations in the scatterplot of regression scores onto size (Figure 7) suggests that the effect of size on shape, although weak, is very similar in the two populations: bigger leaves tend to be slender (‘Campobasso shape’) and smaller ones tend to be wider (‘Busso shape’) (Figure 6, Figure 8). The allometric trajectory is largely aligned with the vector of mean shape differences, as it was somewhat expected since Campobasso has larger leaves. A non-significant test for intercepts also means that there is no support for population differences using the available samples when the effect of size on shape variation is held constant. This is congruent with the result of the permutation test on ‘size-corrected’ shapes (Tables 4–5), which was also non-significant. However, ‘size-corrected’ data slightly increased classification accuracy in the cross-validated DA.

**Table 6:** Allometric shape variation after averaging leaves within trees.

effect	explained	Pillai's trace	F	df	P
slopes (*populations* × *size*)	15.5%	0.269	0.471	18, 23	0.946
intercepts (*populations*)	9.3%	0.523	1.461	18, 24	0.191
*size* only	6.8%	0.624	2.305	18, 25	0.027

Tests for differences between *populations* in slopes and intercepts (MANCOVAs respectively with or without interaction) of allometric trajectories and regression of shape onto centroid size regardless of groups.

## Discussion

What have we learnt from this example study? We start briefly summarizing the main results and their interpretation. After this introductory section, we move to the main topic, the application of GMM in taxonomic botany and the pros and cons of the simplified protocol we have presented.

### a) Phenotypic variation in Q. petraea: do populations and trees differ in leaf size and shape?

These samples were collected to be used as an example of taxonomic comparison using GMM in botany. This is a very narrow aim. Nevertheless, we can draw some preliminary conclusions on the amount and significance of shape and size variation in sessile oak leaves from the Campobasso-Busso area of Molise:

First of all, a very small measurement error showed that the landmarks of sessile oak leaves can be located with precision and images are acquired with accuracy using a simple flatbed scanner. The flatness of leaves in oaks and many other plants and the use of well defined landmarks at the meeting points of veins and other clearly defined structures make them a study material particularly suitable for two-dimensional GMM analyses. However, even apparently easily recognizable landmarks, as the intersection of leaf veins, do not always correspond to biologically homologous regions and must be selected with the uttermost care using the best available knowledge on the study taxon. Indeed, although truly homologous landmarks are often difficult to identify in plants (Jensen, 2003), working on groups of closely related species and taking advantage of specific features with a clear correspondence, as it is often the case with lobes in lobate leaves, can help to discriminate specific vein patterns with accurate homological relationships.

The individual variation in leaf morphology was large. This is likely related to plasticity. Small differences in nutrients and water availability, sun exposure, humidity and other environmental factors which may vary between and within trees can have an effect on leaf blades, vein patterns and leaf contours [56]–[58].

Differences between trees were slightly larger than those within trees. This observation is reasonable in terms of both genetics and environment, as leaves from the same tree share not only identical genes but also to some extent a more homogeneous environment compared to leaves from different trees.

Differences between the two geographic populations were small. This also matches our expectations, as we are comparing samples from neighbouring localities within the same species. The Procrustes ANOVA suggested a modest but nevertheless appreciable variation in size and shape. Size differences were statistically significant even after averaging leaves within trees. This did not happen with shape, but this may be as a result of inadequate statistical power from small samples. The two study populations are characterized by similar environmental conditions but are located in two different valleys of the same mountain (Monte Vairano). The molecular data collected to date indicate a genetic flow between populations in this area and no statistically significant genetic differences [59], which seems to suggest plasticity as the main source of variation between populations. Common garden experiments might be needed to provide a definitive answer to whether differences reflect genetic divergence or plastic responses.

The preliminary and largely exploratory results we have obtained indicate that GMM is truly effective in revealing very small-scale variation. To better understand phenotypic variation in sessile oaks the study will have to be expanded by sampling more localities and a larger range of environmental conditions. Modelling geographic variation in the Italian and European populations of sessile oaks might help to quantify patterns including clines, the occurrence of isolation by distance and the covariation with environmental and genetic factors. An accurate knowledge of heritable phenotypic diversity makes an essential contribution to an effective management of forests and an accurate forecasting of future trends in a rapidly changing environment.

### a) Superimposition methods, shape diagrams and biological interpretations

If the correspondence of landmarks in terms of their biology is a fundamental assumption in taxonomic studies, the method one selects to extract size and shape information from those points and the consequences of that choice on the interpretation of results should be carefully considered. We have described only one of the possible methods to obtain shape variables from landmark coordinates. The GPA has become almost a standard choice in GMM studies and it is the default option in most programs. However this method has no underlying biological model and it is an effective but arbitrary choice to superimpose specimens and separate size and shape. This ‘arbitrariness’ makes variables generated by landmark based GMM methods ‘special’: the relative positions of landmarks after a Procrustes superimposition capture overall shape differences, but cannot be used to say that these differences mostly concern one or the other landmark. A classical example of this problem is the so called ‘Pinocchio effect’ [26]: if Pinocchio's face was compared using Procrustes landmark coordinates before and after a lie made his nose longer, all landmarks would suggest differences and not only those measuring the nose, as the GPA would have spread the variation in nose length across the whole face.

**Figure 9:**
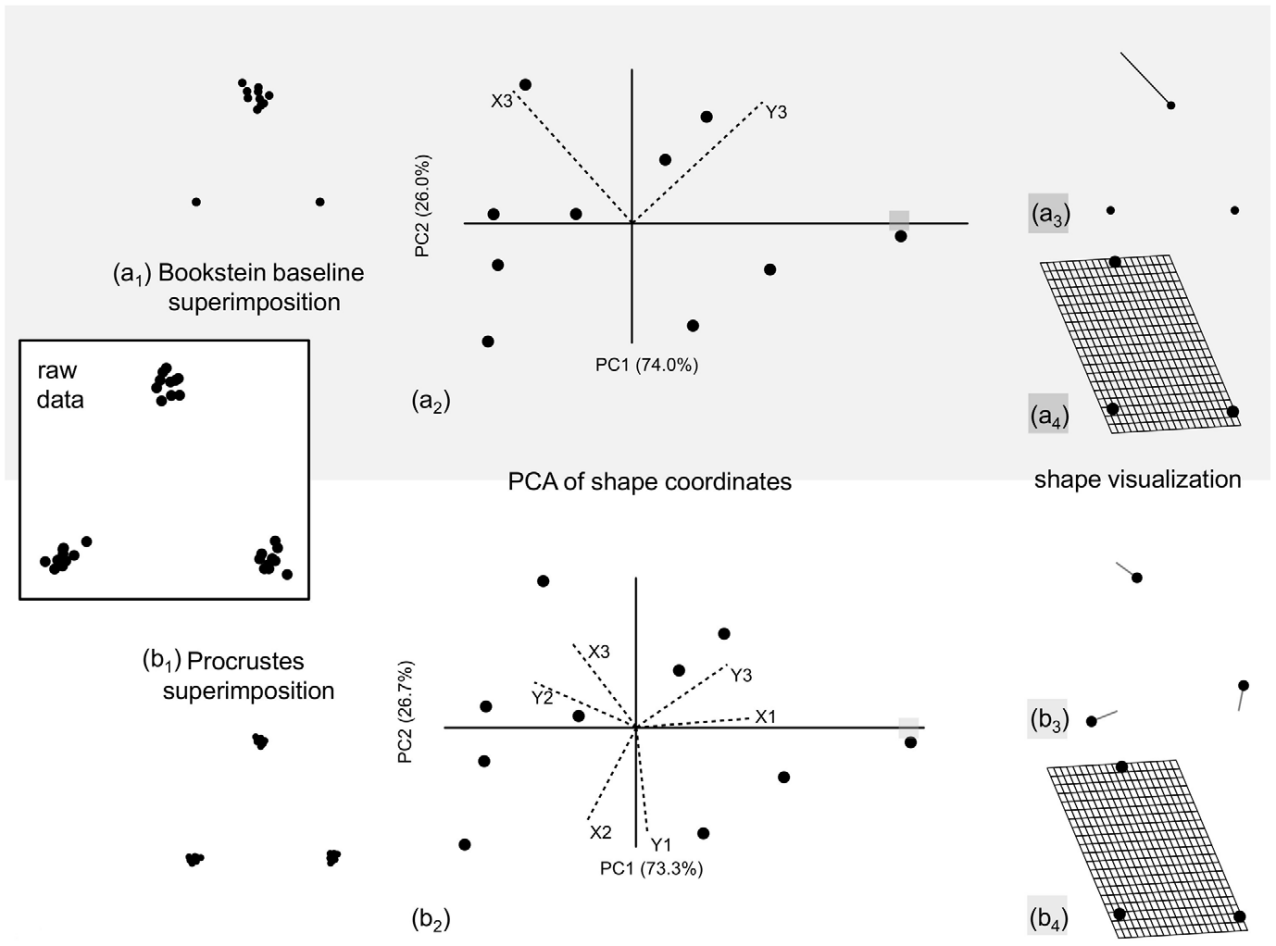
Example of the effect of different superimpositions on the interpretation of results. A set of 10 random triangles (raw data) was superimposed either using Bookstein baseline superimposition (a_1_) or Procrustes (b_1_). Shape coordinates were subjected to PCAs whose results were illustrated using biplots (a_2_, b_2_) showing both the scatterplot of the specimens (filled circles) and the loadings (dotted lines) used to weight the matrix (X1, Y1, etc.) of shape coordinates. Shape variation at the positive extreme of PC1 was visualized magnified four times using either displacement vectors (a_3_, b_3_) or TPS grids (a_4_, b_4_).

Shapes are the same regardless of whether and how they have been superimposed. The superimposition is only an expedient to generate shape variables for quantitative analyses. Shapes are not a function of the superimposition, but landmark coordinates are. Different superimpositions generally produce very similar shape distances for the type of small variation which is found in most biological datasets (e.g., [38], p. 283–287). However, if superimposed configurations are examined, variation around specific landmarks could look remarkably different depending on whether one has used a GPA or another method. For instance, using a baseline superimposition [28] on a sample of random triangles, variation in specimen size and position is removed by simply translating, rotating and rescaling all specimens until two landmarks, selected as a baseline, coincide (Figure 9a_1_). This leaves only one landmark, the one not used as a baseline, to account for all shape differences. If the same sample is Procrustes superimposed, shapes are unmodified but variation is spread across all landmarks (Figure 9b_1_). Performing a PCA on shape coordinates will produce in both cases only two PCs with non-zero variance. Generally, the scatterplot and therefore also the shape distances among specimens will be very similar (Figure 9a_2_, 9b_2_). However, diagrams with superimposed configurations, displacement vectors and PC loadings will be different and suggest rather dissimilar interpretations: using Bookstein baseline, it appears as if there is only one landmark that ‘moves’ to different positions (Figure 9a_3_) and accounts for all shape differences; with Procrustes, all landmarks ‘move’ (Figure 9b_3_) and contribute to a smaller or larger extent to total shape variation. An obvious question then is what method is producing the most accurate outcome to describe shape variation. The answer is both, as long as results are integrated over the space of the whole landmark configuration; and none, if changes are interpreted as occurring at specific landmark locations. The TPS grids follow the first approach and correctly demonstrate that shape variation is the same regardless of the superimposition (Figure 9a_4_, 9b_4_). Displacement vectors, PC loadings, the variance around one or the other landmark, in contrast, all use information dependent on the choice of the superimposition and are therefore potentially misleading. Neither landmark 3 only varies in the precise direction shown in Figure 9a_3_ nor all of them vary by about the same amount but in different directions, as in Figure 9b_3_. The direction of variation is the shearing and contraction/expansion of the whole space of the landmark configuration (Figure 9a_4_, 9b_4_) and the magnitude of the change is the shape distance between the reference and the target, which respectively correspond, in Figure 9a_2_, 9b_2_, to the origin of the axes (the sample mean) and the positive extreme of PC1 and it is about the same regardless of the superimposition.

The ‘arbitrariness’ of the choice of the superimposition has another important implication. One cannot associate the digitizing error to specific landmarks, once specimens have been superimposed. Even if there was only one landmark with very low precision, as in the case of the ‘Pinocchio effect’, the Procrustes superimposition would spread the error across the whole configuration. Using a baseline superimposition this apparent paradox is even more evident as, if the low precision landmark was one of the two two defining the baseline, it would show no error at all. Thus, only by repeating the digitization on the same image of a single specimen without superimposing the replicas, one might get some clues about whether one or the other landmark has a larger error in that specimen.

### b) 'TPS shape variables'

TPS deformation grids without displacement vectors (Figure 8; Figure 9a_4_, 9b_4_) help to describe shape variation in a way that is independent of the superimposition method [26]. The grids are computed using only information on differences in compression/dilation, shear and localized shape changes. These are components of shape variation that are unaffected by scaling, rotation and translation. Because it is variation in scaling, rotation and translation that is standardized in different ways using different superimpositions, the TPS produces, in a sense, a superimposition-free visualization.

The TPS, however, is not a biological model of shape change. It is an interpolating that is expressing changes in the relative positions of the landmarks as a smooth deformation of the entire space and has been originally developed in the context of the physics of thin metal sheets. It is therefore unreasonable to expect the tissue in between landmarks to be modified exactly as described by TPS deformation. This procedure, whose rigorous description is found in Dryden and Mardia [38], Bookstein [53] and in more accessible terms in Zelditch et al. [26], just generates a ‘picture’. Briefly and informally, the intuition behind the TPS is that by minimizing the energy required for bending shapes one into the other, one can derive a set of orthogonal vectors of coefficients called principal warps. Using the principal warps, whose computation relies only on the landmarks, one can predict changes in any region of the sheet, a little bit as one could use regression coefficients to predict the dependent variable for values of the independent one where no observations were available. If on the sheet there is a rectangular grid or an outline, as the one with the veins and contours of the oak leaf used in our study, then the TPS will predict the way the lines are deformed when a reference shape is warped to match a target configuration. Grids and outlines might help to describe shape differences and suggest where interesting changes are happening, but they have to be cautiously interpreted as they may not be accurate.

As in a PCA, variables are combined by projecting the observations on the eigenvectors, principal warps can be used also as a new set of axes on which to project shape coordinates. The result is a linear combination of the original variables called partial warps (for the localized shape changes) and uniform components (for changes which occur the same way everywhere and therefore leave the grid lines parallel – e.g., a uniform compression of the grid). Partial warps and uniform components provide all together the same information as the original shape coordinates or their PCs. This is easily tested by comparing shape distances based on the shape coordinates, their PCs or the partial warps plus the uniform components. The shape data are the same and it is only the ‘point of view’ by which we are looking at them that has changed: in a PCA we want to see the main directions of variation in the whole sample; with the partial warps and uniform components we rotate the axes to find the directions which require the smallest amount of energy for bending the grids. In terms of practical use, however, there is an important distinction to be made. The ‘directions’ (i.e., the eigenvectors) in a PCA are based on the way landmarks vary and covary in all specimens of a sample. For instance, in our study, PC1 showed that the main axis of variation has something to do with the relative elongation of the leaf blade and narrowing of the leaf base, which is informative. In contrast, the ‘directions’ of the TPS (i.e., the principal warps) are based only on the relative positions of landmarks in a specific reference configuration (generally the sample mean) and are derived to minimize a quantity, the energy required to bend a thin metal sheet, which is meaningless in a biological context. This is why, unlike PCs that can be used for scatterplots and sometimes as a subset of them to reduce dimensionality, partial warps and uniform components only make sense if used as a whole [60]. For the same reason, even if some of programs developed in the early days of GMM may still include univariate or bivariate tests, scatterplots and visualizations of partial warps and uniform components, these are highly unlikely to be of any use and only the multivariate use of all partial warps and uniform components results should be taken into consideration.

In conclusion, PCs are generally a better choice as shape variables for statistical analyses. However, users do not have to forget that PCs are derived only to maximize total sample variance regardless of groups or any other factor (environmental correlates, phylogeny etc.). Shape is inherently multivariate and this generally means that all shape variables (or an adequate number of the first PCs selected using a valid and explicit criterion) must be used for modeling shape variation. It is most unlikely that they can be analysed one at a time to test group differences, in univariate regressions, to measure the fit of each of them to a phylogenetic tree and so on. Their use as phylogenetic characters is also questionable [61] and even the newest solutions proposed might be in need of substantial improvements, as suggested by Felsenstein's cautionary statement (“we can do better”) in a recent online discussion forum (https://stat.ethz.ch/pipermail/r-sig-phylo/2010-November/000825.html) and the ephemeral fate of all those put forward in the past two decades [5-59–60].

### c) A hierarchy of differences: assumptions and interpretation of the Procrustes ANOVA

In taxonomy statistical testing mostly concerns group differences. Groups generally are populations within a species or different species. A single individual, however, is itself variable, as it may have many leaves or many flowers, for instance. One may first want to test whether differences between individuals (e.g., trees) in a group are larger than the natural variation within an individual and, even more crucially, one needs to demonstrate that measurement error is negligible. For this purpose, we adapted a protocol originally developed for studies of bilateral asymmetry in Procrustes shape data [25] and later implemented in MorphoJ [24]. To keep the analysis simple and easy to replicate for taxonomists who might not have a strong background in statistics, we used the isotropic model described by Klingenberg et al. [25]. This enables any user to easily correct manually the F ratio for *populations* using *trees* as a random effect. The isotropic, however, model assumes a similar amount of variation around each landmark in any direction. As there is no directional variation, one is only considering the magnitude of the effect being tested by summing up its univariate sum of squares. Computations are easier but the assumption used is rather restrictive and not unlikely to be violated [25]. If this happens, P values may become unreliable.

Klingenberg et al. [25] advised to use permutation tests in small samples, when deviations from normality are suspected, and a MANOVA approach, if one wants to avoid the isotropic assumption. None of these methods has yet been implemented, unfortunately, in a user-friendly format applicable to designs other than that of the original Procrustes ANOVA and the study of symmetric structures. Performing the computations manually using the simple isotropic parametric model is the only simple option we could find, although it is a rather suboptimal one and results must be interpreted with the greatest caution. For instance, in our study measurement error was tiny (< 0.1%) and this made us confident that we were on the safe side. The effect of *populations* was also statistically significant, but F was not particularly large and the percentage of explained sum of squares was small (≈ 3–6%). After averaging leaves within trees, *populations* were no longer statistically significant for shape and specimens were inaccurately classified in the DA. Overall, therefore, our conclusion was that there might be small differences between populations, but the evidence for them is weak and larger samples will be needed to obtain robust results.

Using a hierarchical design for testing group differences provides answers on the degree of variation at different levels and it does so by taking into consideration the structure of the data. Leaves of the same tree are more likely to be similar, as they share the same genes and grew up in the same environment. This means that they are not independent observations and represent to a certain degree pseudoreplicates. If we had not taken that into account (which we did using the hierarchical design), we might have violated a basic assumption of most statistical tests: the independence of data. Consequences of this violation can be serious, as degrees of freedom as well as the error term of regression models might be incorrectly computed [62]. Tests using resampling statistics may become unreliable too, because both permutations and bootstraps assume that each observation within a factor is like every other one, which is clearly not true if there are pseudoreplicates. Autocorrelation, the similarity between observations as a function of the time, space or other factors, can occur also because of spatial distribution and (phylo-) genetics: for instance, trees living in close proximity are more likely to be similar than trees which are further apart, and sister species are more likely to have characters in common between them than with any other species. Models addressing various aspects of these issues can be found in the literature on spatial data analysis (e.g., [62], [63]) and comparative methods (e.g., [64]–[66]). As methods are developed and more software becomes available, taking into account different sources of non-independence in the data will hopefully become easier in univariate and especially multivariate analyses.

### d) Testing taxonomic groups: why results of DAs should be interpreted with caution and classification tables must always be cross-validated

When there is a single grouping variable, DA is probably the most common method for testing differences and predicting groups. It was developed by Fisher [67], Mahalanobis and other statisticians in the '30s and has been widely used in biology ever since. It is a well known technique and does not require detailed explanations. In the Material and Methods we have given introductory references about DA in general and more specifically in GMM. In this section we emphasize two related aspects of the method, which often mislead inexperienced users.

The first one is that DA tests differences, predict group affiliation and provide scatterplots in a data space which is no longer the same as the one of the original variables. In our specific case, this means that we are no longer in the shape space generated by the Procrustes superimposition (or in its projection into a Euclidean space). Within samples, variance is squeezed in all directions around group means to make it circular. Between samples, the direction of largest mean differences is found to project the data on axes which best discriminate groups. In this transformed space distances between observations are called Mahalanobis distances and are scaled in a way such that a unit distance corresponds to one standard deviation. Using distances between individual observations and sample means, the relative probability of a specimen to belong to one or the other group can be computed. Using a multivariate normal distribution, one can also estimate the absolute probability (called typicality) of a specimen to belong to the group whose mean is the closest. This allows to say not only which group the specimen is classified into, using that set of predictors, but also if it is within or outside the range of variation typical for that group [35], [48]. If it is outside, there is the possibility that it actually does not belong to any of the available a priori groups, a common finding especially when fossils are included [48]. One can produce scatterplots of specimens in the transformed space of the DA and also use the statistical properties of this space to test the significance of group differences. The test and its assumptions (independence of observations, multivariate normality, homoscedasticity) are the same as in a one way MANOVA. As in other parametric methods, assumptions should be carefully considered because, despite a relative robustness to, for instance, moderate violations of normality, results may be inaccurate. Heterogeneous sample sizes, small samples and highly multivariate data may be particularly problematic. It is a situation which is not uncommon in taxonomic studies [2] and something that makes statistical inference potentially unreliable and the tests of assumptions difficult. A DA can still be done but it might be more for descriptive and classificatory purposes than for testing [35]. In our example study, there was no evidence for violations of homoscedasticity (tested using Box's M, results not shown), but multivariate normality was not tested because of the relatively small samples and large number of variables. Permutation tests, which do not assume normality, were congruent with parametric ones and this increased our confidence in the accuracy of results.

A second important and often neglected issue is that DA tends to overfit the data [68], [69]. To put it simply, by using a data space derived in order to improve classification accuracy, a DA tend to force differences to appear even when they are negligible or absent. This is easily appreciated by creating a set of variables made of random numbers divided into a few arbitrary groups on which one performs a DA. The larger the number of predictors relative to sample size, the higher the classification accuracy and the better the discrimination of groups in scatterplots despite the absence of real differences [50]. Because GMM tends to generate large number of variables (little less than twice or three times the number of landmarks in respectively two- and three-dimensional analyses) and that is likely to become larger as the use of semilandmarks on curves and surfaces becomes more widespread [5], overfitting is a serious problem. Dimensionality reduction (e.g., [70]) may help but comes at the cost of a loss of information and does not fully address the issue. There is, fortunately, a fairly straightforward way to assess the consequences of the problem on a specific dataset. One has to cross-validate group predictions, as we showed in our example study. For instance, in the hypothetical case we made using random numbers and arbitrary groups, the percentages of correctly classified specimens drop to about random chance (i.e., 50% in a balanced sample with two groups) after cross-validation. This is why cross-validation should be customary and only cross-validated classification tables should be discussed. When differences are statistically significant and cross-validated classification accuracy is high, the data support the occurrence of taxonomic differences. However, taxonomists know that this is only a small piece of evidence to establish taxonomic groups and it has to be complemented with other sources of information, including ecology and genetics, to make meaningful statements about subspecific, specific or supraspecific status.

### e) Shape differences controlling for allometry

Size is often considered more evolutionary labile than shape [20], [21], [71]–[75]. A taxonomist may want to assess whether differences in shape actually simply mirror size differences because of allometry. If that happens, one has only one independent piece of evidence for group differences: size with its allometric effect on shape. Indeed, GMM using the Procrustes superimposition efficiently separates size and shape, but does not remove the covariation between these two components. A classical MANCOVA model is a simple way to test the effect of size on shape when comparing groups. Zelditch et al. [26] provide a clear description of this method and offer a non-parametric alternative for testing slopes and intercepts. Mitteroecker et al. [76] discuss a different framework to compare allometries, but did not provide the software needed to perform the analysis. MorphoJ has implemented an extension of the MANCOVA model to compute ‘size-corrected’ data that can be used as a new set of shapes for analyses of ‘non-allometric’ variation. The ‘size-correction’ strongly depends on the assumption of parallel allometries. An extensive example of a taxonomic analysis on ‘size-corrected’ data is found in Elton et al. [51], who also suggested a modified version of the MANCOVA model to remove the effect of evolutionary allometry, as an alternative to the standard protocol to control within group allometric variation. The usefulness of this alternative, and its relationship with Gould's [77] criticisms to the traditional MANCOVA model in an evolutionary context, will have to be explored in future studies.

Finally, users should be aware that in large samples small but inconsequential differences in slopes could be significant because of the high statistical power. This was likely the case in a study by Cardini and Elton [73] on a sample of more than 1300 individuals. Despite significance, separate and parallel allometric trajectories fit their data about equally well, as indicated by the percentage of variance explained which was very similar (respectively 43.5% and 41.6%) and more than 4 times larger than the amount (9.8%) explained by a single regression line regardless of groups. In cases such as this, the taxonomist might have a reason to argue that data can be ‘size-corrected’ in spite of significant slopes, as results are unlikely to be appreciably affected by the small violation of this assumption.

### Conclusions

The series of analyses we have described and discussed in this paper represents a simplified framework for taxonomic studies on group differences in botany and other disciplines. It takes its inspiration from and expands the example by Rohlf et al. [78], discussed in Rohlf's [60] seminal paper on the biological interpretation of shape variables. It is a classical application to shape data of methods commonly used in traditional multivariate morphometrics [2], [35]. Its main aim is to provide taxonomists with little or no experience in GMM with a clear, simple and easy to follow step-by-step protocol that may help them to familiarize with the method avoiding some of the most common pitfalls. Beginners might then be less intimidated by the often difficult GMM literature and may become interested in exploring the usefulness and potential of GMM in their work. Reading the excellent introductory book by Zelditch et al. [26] and perusing the main morphometric website http://life.bio.sunysb.edu/morph/index.html could be the next step to learn more about theory, applications and the variety of software available besides those few programs and methods described in our study. A list of recommendations on the main methodological issues outlined in the Discussion is available in the Appendix S1; in addition, Appendix S2 provides information on the datafiles available as supplentary information: a worked out MorphoJ project (MorphoJ Project S1), the leaf outline (MorphoJ Outline S1), the raw data (MorphoJ Raw Data S1), the averaged tree shape data (TPSRegr Averaged Tree Data S1) and the dummy variables for tests in TPSRegr (TPSRegr Dummy Data S1).

## Supporting Information

Appendix S1 Recommendations on methodology.
(DOC)

Appendix S2 Information on data files.
(DOC)

MorphoJ Project S1 A worked out MorphoJ project.
(MORPHOJ)

MorphoJ Outline S1 Leaf outline.
(TXT)

MorphoJ Raw Data S1 Rawdata in .nts format.
(NTS)

TPSRegr Averaged Tree Data S1 The averaged tree shape data for tests in TPSRegr.
(NTS)

TPSRegr Dummy Data S1 The dummy variables for tests in TPSRegr.
(NTS)
