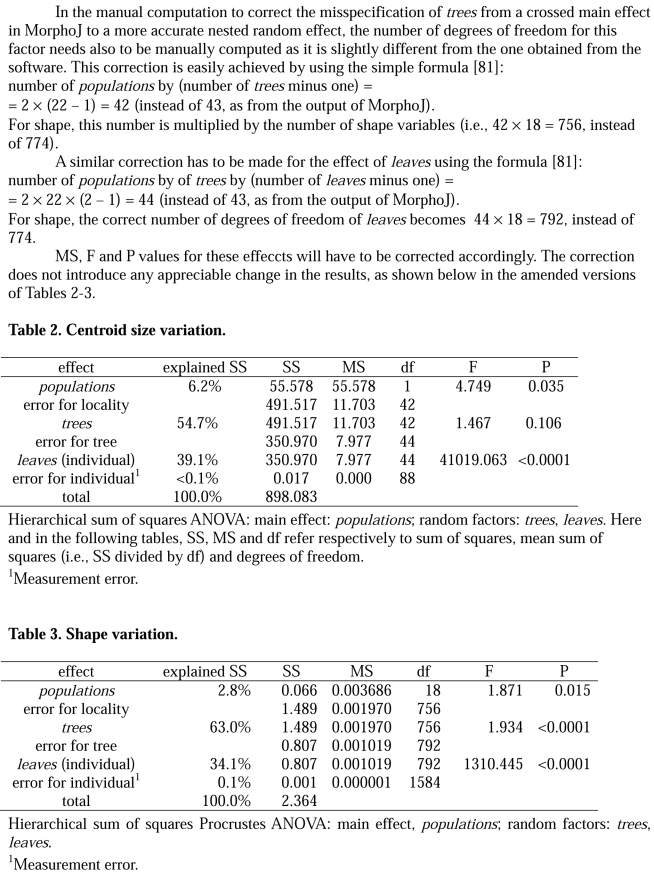# Correction: Leaf Morphology, Taxonomy and Geometric Morphometrics: A Simplified Protocol for Beginners

**DOI:** 10.1371/annotation/bc347abe-8d03-4553-8754-83f41a9d51ae

**Published:** 2012-03-07

**Authors:** Vincenzo Viscosi, Andrea Cardini

There was a computational error in the degrees of freedom, affecting Tables 2 and 3. The entire correction can be viewed here: 

**Figure:**