# Supplementary material for: Leaf Morphology, Taxonomy and Geometric Morphometrics: A Simplified Protocol for Beginners
Source: PLoS One. 2011 Oct 3;6(10):e25630. doi: 10.1371/journal.pone.0025630 (PMC3184990; doi:10.1371/journal.pone.0025630)
Supplement: Appendix S1 — Recommendations on methodology. (DOC) [file pone.0025630.s001.doc]

Appendix 1

*Recommendations on the main methodological issues outlined in the Discussion:*

*a) Superimposition methods, shape diagrams and biological interpretations*

- use shape diagrams to interpret results in terms of variation *over the whole configuration* of landmarks;
- be *careful* when *displacement vectors* are used and more generally any time the reference and target shape are shown superimposed;
- if it helps, remove displacement vectors and show reference and target shapes one next to the other: this will almost inevitably force you to describe differences in terms of spatially intergrated shape changes*;
- never interpret coefficients and more generally variation at specific landmarks independently of others.

*b) 'TPS shape variables'*

- take advantage of the effectiveness of TPS diagrams, but be careful not to over-interpret variation, especially if distant from the nearest landmarks, because everything except the landmarks themselves is an interpolation (i.e., a 'mathematical guess');
- never use subsets of partial warps and uniform components, as they are derived to minimize bending energy, which is a meaningless concept in biology;
- generally use PCs of shape coordinates as variables, as they are more flexible and remove any redundancy in the data (not only the loss of degrees of freedom after the GPA, but also the one due to small sample size or the mathematical manipulation of semilandmarks – [5,26];
- if a dimensionality reduction is unavoidable, use an explicit criterion to determine how many of the first PCs are enough to accurately summarize shape variance (e.g., [79]);
- avoid using the term relative warp analysis (a PCA on the matrix of partial warps and uniform components), because it is generally redundant and unfamiliar to scientists outside the field of GMM.

*c) A hierarchy of differences: assumptions and interpretation of the Procrustes ANOVA*

- perform the Procrustes ANOVA using the isotropic model, but be cautious in interpreting results and clearly state its limits
- if possible repeat the test with less restrictive models (MANOVA using permutations); nota bene: if there are no leaves or other multiple structures to sample from the same specimen (as, for instance, in most zoological taxonomic studies), one will simply need to include the individual and *populations* factor to test groups and measurement error with all computations done directly in MorphoJ using both the isotropic and the less restrictive non-isotropic model.
- use a balanced design, if possible, as computations and interpretations are easier and unequal sample sizes tend to make P values less accurate [80];
- be aware of autocorrelation especially when analysing data with spatial or phylogenetic structure.

*d) Testing taxonomic groups: why results of DAs should be interpreted with caution and classification tables must always be cross-validated*

- in a test of differences between two or more groups (as well as in regressions etc.), estimates of the variation explained by the factor being tested are often available or easy to compute: they provide useful complimentary information to significance values as they say how well that factor fits the data**;
- check the assumptions of DAs (and other models used for statistical inference) whenever possible;
- increase sample size, use balanced samples and, when sample sizes are very heterogeneous, check that results are robust to the exclusion of the smallest/largest samples;
- consider the possibility of dimensionality reduction, but be careful and use an explicit criterion to do it;
- be cautious with the interpretation of results especially if a drop in classification accuracy after cross-validation suggests a serious problem of overfitting (i.e., 'over-optimistic' results which will affect not only the classification, but also the DA/CVA scatterplots).

*e) Shape differences controlling for allometry*

- if the simple MANCOVA model is used, always test the interaction term (i.e., differences in slopes) before doing a 'size-correction';
- be particularly cautious when the model is applied in a comparative context, where more appropriate 'corrections' might be available and data may not be fully independent because of phylogeny.

*We did not make this choice to make a simple and direct use of the graphical output of MorphoJ. However, images can be saved in MorphoJ as svg files and easily edited using a vector graphics software as Inscape ([www.inkscape.org/](http://www.inkscape.org/)) to change the visualization.

**Sokal & Rohlf [81] in their chapter on the nested ANOVA show how to go one step forward compared to our simple suggestion of computing percentages of sum of squares explained by different factors. One may want to know how much variance is added by an effect once all those below it have been taken into account (i.e., the variance explained by them has been removed). This requires estimating variance components and it is achieved by computing the difference between the mean sum of squares (MS) of that effect and the one of the effect immediately below it divided by the sample size for that effect. For instance, if one wants to know the variance component of *leaves* in the ANOVA for size, he/she has simply to subtract the error MS from the *leaves* MS and divide the difference by the number of replicas for each leaf, i.e. (8.162 – 0.000) / 2 = 4.081. The same could be done for *trees* using the difference between their MS and that of *leaves* divided by four (i.e., two leaves per tree and two replicas per leaf), thus obtaining (11.431 - 8.162) / 4 = 0.817. Finally, one could also compute the variance component of the main effect of *populations*, which is its MS minus the MS of *trees* divided by how many 88 leaves per population including replicas, which is (55.578 - 11.431) / 88 = 0.502. Expressed in relative terms, as percentages of the total variance in those factors (i.e., including the error term, 0.000 + 4.081 + 0.817 + 0.502 = 5.400), the error term accounts for virtually no variance, *leaves* for 75.6%, *trees* for 15.1% and *populations* for the remaining 9.3%. If the same computations were performed for shape (which requires multiplying the denominator by the number of variables, i.e. 18), one would obtain the following variance components: error, 1.8%; *leaves*, 67.2%; *trees*, 28.4%; *populations*, 2.6%. Thus, in both size and shape most of the variation occurs among leaves, but trees add 15% (size) to almost 30% (shape) extra variation and geographic populations add another 3% (shape) to almost 10% (size). This indicates that individual leaf differences dominate the pattern of variation in our samples and also suggests that trees within a population on average tend vary more in shape than size, but differences in size between populations are more consistent than those in shape, therefore producing appreciable size variation between localities.

In this example, variance components were computed for the random factors as well as for the main effect. This is unorthodox as variance components are generally estimated only for the random factors one is trying to control for. However, in an observational taxonomic study, knowing how much variance is accounted for by all factors can be informative, as the relative magnitude of population differences compared to the variation found among trees and leaves is another interesting aspect of the the biology of the study group.

Readers interested in learning more on variance components will find a general introduction in Macdonald ([80] - McDonald JH (2009) Handbook of Biological Statistics, 2nd ed. Sparky House Publishing, Baltimore (freely available online at http://udel.edu/~mcdonald/statintro.html or as pdf file at http://www.lulu.com/items/volume_66/7719000/7719172/1/print/STATPDF.PDF)) and more information, including the general formula for unequal sample sizes, in Sokal & Rohlf [81], as well as in more advanced statistical textbooks, which may also cover the theory of alternative methods to estimate variances and their confidence intervals.
