# Supplementary material for: Leaf Morphology, Taxonomy and Geometric Morphometrics: A Simplified Protocol for Beginners
Source: PLoS One. 2011 Oct 3;6(10):e25630. doi: 10.1371/journal.pone.0025630 (PMC3184990; doi:10.1371/journal.pone.0025630)
Supplement: Appendix S2 — Information on data files. (DOC) [file pone.0025630.s002.doc]

Appendix 2

*Information on data available as supplementary information*

Raw data are provided in NTS format (data_S1.nts). This file can be imported in MorphoJ, used to extract classifiers and perform the analyses described in the paper. From MorphoJ variables can be easily exported to PAST (do not forget to rename ID with label in the MorphoJ .txt file !), spreadsheets like Gnumeric (just drag the MorphoJ .txt file in the Gnumeric window) or other programs. A file with a leaf outline (outline_S1.txt) is also provided which can be directly imported in MorphoJ and used for the visualization.

A worked out MorphoJ project (project_S1.morphoj) is also available.

(Nota bene: 1) it has to be opened from within the program as a project file; 2) *trees* could not be specified as a random effect and the appropriate correction must be done manually as explained in the paper).

NTS files (data_S2.nts, data_S3.nts) for testing slopes and intercepts in TPSRegr are provided for convenience but can be easily created from the original raw datafiles following the detailed example in the program help file.
